# Supplementary material for: Rapid changes in risk attitudes originate from Bayesian inference on parietal magnitude representations
Source: Nat Commun. 2026 Sep 10;17:10703. doi: 10.1038/s41467-026-77402-6 (PMC13658247; doi:10.1038/s41467-026-77402-6)
Supplement: Supplementary file 2 [file 41467_2026_77402_MOESM2_ESM.pdf]

nature portfolio

Corresponding author(s): Gilles de Hollander

Last updated by author(s): Oct 21, 2024

Reporting Summary

Nature Portfolio wishes to improve the reproducibility of the work that we publish. This form provides structure for consistency and transparency in reporting. For further information on Nature Portfolio policies, see our Editorial Policies and the Editorial Policy Checklist.

Statistics

For all statistical analyses, confirm that the following items are present in the figure legend, table legend, main text, or Methods section.

n/a	Confirmed	
☐	☒	The exact sample size (*n*) for each experimental group/condition, given as a discrete number and unit of measurement
☐	☒	A statement on whether measurements were taken from distinct samples or whether the same sample was measured repeatedly
☐	☒	The statistical test(s) used AND whether they are one- or two-sided<br>*Only common tests should be described solely by name; describe more complex techniques in the Methods section.*
☒	☐	A description of all covariates tested
☒	☐	A description of any assumptions or corrections, such as tests of normality and adjustment for multiple comparisons
☐	☒	A full description of the statistical parameters including central tendency (e.g. means) or other basic estimates (e.g. regression coefficient) AND variation (e.g. standard deviation) or associated estimates of uncertainty (e.g. confidence intervals)
☐	☒	For null hypothesis testing, the test statistic (e.g. *F*, *t*, *r*) with confidence intervals, effect sizes, degrees of freedom and *P* value noted<br>*Give P values as exact values whenever suitable.*
☐	☒	For Bayesian analysis, information on the choice of priors and Markov chain Monte Carlo settings
☐	☒	For hierarchical and complex designs, identification of the appropriate level for tests and full reporting of outcomes
☒	☐	Estimates of effect sizes (e.g. Cohen's *d*, Pearson's *r*), indicating how they were calculated

Our web collection on statistics for biologists contains articles on many of the points above.

Software and code

Policy information about availability of computer code

Data collection

Data was collected using Psychopy as wrapped in exptools2
https://github.com/VU-Cog-Sci/exptools2

All code that was used to present the experiment to participants can be found on
https://github.com/ruffgroup/risk_experiment/tree/master/experiment

Data analysis

Data was analyzed in Python.

For the numerical receptive field model fitting we used our package braincoder
https://braincoder-devs.github.io/

For the cognitive models we used our package bauer
https://github.com/ruffgroup/bauer/tree/main

as well as the package bambi
https://bambinos.github.io/bambi/index.html

All analysis code can be found on
https://github.com/ruffgroup/risk_experiment/

For manuscripts utilizing custom algorithms or software that are central to the research but not yet described in published literature, software must be made available to editors and reviewers. We strongly encourage code deposition in a community repository (e.g. GitHub). See the Nature Portfolio guidelines for submitting code & software for further information.

Data

Policy information about availability of data

All manuscripts must include a data availability statement. This statement should provide the following information, where applicable:

- Accession codes, unique identifiers, or web links for publicly available datasets
- A description of any restrictions on data availability
- For clinical datasets or third party data, please ensure that the statement adheres to our policy

The raw fMRI data generated in this study have been deposited on OpenNeuro under accession code ds007508 and are publicly available at https://openneuro.org/datasets/ds007508150. The behavioural data from the symbolic (Arabic-numeral) risk task are publicly available on figshare (https://doi.org/10.6084/m9.figshare.31400430)

Research involving human participants, their data, or biological material

Policy information about studies with human participants or human data. See also policy information about sex, gender (identity/presentation), and sexual orientation and race, ethnicity and racism.

Reporting on sex and gender	We report self-reported gender. We did not perform any gender-specific analysis, because we do not expect any gender effects and the n was too low to have sufficient power for such analyses.
Reporting on race, ethnicity, or other socially relevant groupings	We do not report on race, ethnicity or other socially relevant groupings beside age.
Population characteristics	See above
Recruitment	Participants were recruited via the University of Zurich participant database; standard self-selection biases for volunteer neuroimaging samples apply (MR-compatible, no neurological/psychiatric history), which is unlikely to affect the within-subject perceptual mechanisms of interest.
Ethics oversight	Cantonal Ethics Committee Zurich

Note that full information on the approval of the study protocol must also be provided in the manuscript.

Field-specific reporting

Please select the one below that is the best fit for your research. If you are not sure, read the appropriate sections before making your selection.

☐ Life sciences ☒ Behavioural & social sciences ☐ Ecological, evolutionary & environmental sciences

For a reference copy of the document with all sections, see nature.com/documents/nr-reporting-summary-flat.pdf

Behavioural & social sciences study design

All studies must disclose on these points even when the disclosure is negative.

Study description	The study design is quantitative: two pre-registered-style within-subject experimental studies (a 3T/7T fMRI study and a behavioural study) using incentive-compatible binary risky choice with computational (Bayesian) modelling of behaviour and neural data.
Research sample	The research sample is described with demographics (fMRI: n = 30, 14 female, ages 20-34; behavioural: n = 58, 31 female, mean 24.6, range 20-41), recruited from the local University of Zurich participant pool; this is a convenience sample typical for psychophysical/neuroimaging studies and is not intended to be population-representative, which is appropriate for the within-subject mechanistic questions addressed.
Sampling strategy	Sampling was by convenience from the local participant pool. No formal power analysis was performed to predetermine sample size; the sample sizes (n = 30; n = 58) are in line with, or larger than, comparable within-subject psychophysical/neuroimaging studies and are supported by the parameter- and model-recovery analyses demonstrating adequate sensitivity.
Data collection	The data-collection procedure and instruments are described (MRI scanners and sequence parameters; Avotec goggle stimulus presentation; button-box responses). Only the participant and experimenter were present; the task was computer-administered and the experimenter was not blinded as the manipulation is within-subject and condition is defined per trial by the program (no between-group allocation).
Timing	Data was collected between September 2020 and February 2021
Data exclusions	No exclusion.

Non-participation	One participant dropped out after the first (out of four) sessions. She preferred to not go into a MRI scanner again.
Randomization	There were no experimental groups; all manipulations (presentation order, stake size) were within-subject and randomised across trials by the presentation software, so group allocation is not applicable.

Reporting for specific materials, systems and methods

We require information from authors about some types of materials, experimental systems and methods used in many studies. Here, indicate whether each material, system or method listed is relevant to your study. If you are not sure if a list item applies to your research, read the appropriate section before selecting a response.

Materials & experimental systems

n/a	Involved in the study
☐	☐ Antibodies
☐	☐ Eukaryotic cell lines
☐	☐ Palaeontology and archaeology
☐	☐ Animals and other organisms
☐	☐ Clinical data
☐	☐ Dual use research of concern
☐	☐ Plants

Methods

n/a	Involved in the study
☐	☐ ChIP-seq
☐	☐ Flow cytometry
☐	☒ MRI-based neuroimaging

Antibodies

Antibodies used	*Describe all antibodies used in the study; as applicable, provide supplier name, catalog number, clone name, and lot number.*
Validation	*Describe the validation of each primary antibody for the species and application, noting any validation statements on the manufacturer's website, relevant citations, antibody profiles in online databases, or data provided in the manuscript.*

Eukaryotic cell lines

Policy information about cell lines and Sex and Gender in Research

Cell line source(s)	*State the source of each cell line used and the sex of all primary cell lines and cells derived from human participants or vertebrate models.*
Authentication	*Describe the authentication procedures for each cell line used OR declare that none of the cell lines used were authenticated.*
Mycoplasma contamination	*Confirm that all cell lines tested negative for mycoplasma contamination OR describe the results of the testing for mycoplasma contamination OR declare that the cell lines were not tested for mycoplasma contamination.*
Commonly misidentified lines (See ICLAC register)	*Name any commonly misidentified cell lines used in the study and provide a rationale for their use.*

Palaeontology and Archaeology

Specimen provenance	*Provide provenance information for specimens and describe permits that were obtained for the work (including the name of the issuing authority, the date of issue, and any identifying information). Permits should encompass collection and, where applicable, export.*
Specimen deposition	*Indicate where the specimens have been deposited to permit free access by other researchers.*
Dating methods	*If new dates are provided, describe how they were obtained (e.g. collection, storage, sample pretreatment and measurement), where they were obtained (i.e. lab name), the calibration program and the protocol for quality assurance OR state that no new dates are provided.*

☐ Tick this box to confirm that the raw and calibrated dates are available in the paper or in Supplementary Information.

Ethics oversight	*Identify the organization(s) that approved or provided guidance on the study protocol, OR state that no ethical approval or guidance was required and explain why not.*

Note that full information on the approval of the study protocol must also be provided in the manuscript.

Animals and other research organisms

Policy information about studies involving animals; ARRIVE guidelines recommended for reporting animal research, and Sex and Gender in Research

Laboratory animals	*For laboratory animals, report species, strain and age OR state that the study did not involve laboratory animals.*
Wild animals	*Provide details on animals observed in or captured in the field; report species and age where possible. Describe how animals were caught and transported and what happened to captive animals after the study (if killed, explain why and describe method; if released, say where and when) OR state that the study did not involve wild animals.*
Reporting on sex	*Indicate if findings apply to only one sex; describe whether sex was considered in study design, methods used for assigning sex. Provide data disaggregated for sex where this information has been collected in the source data as appropriate; provide overall numbers in this Reporting Summary. Please state if this information has not been collected. Report sex-based analyses where performed, justify reasons for lack of sex-based analysis.*
Field-collected samples	*For laboratory work with field-collected samples, describe all relevant parameters such as housing, maintenance, temperature, photoperiod and end-of-experiment protocol OR state that the study did not involve samples collected from the field.*
Ethics oversight	*Identify the organization(s) that approved or provided guidance on the study protocol, OR state that no ethical approval or guidance was required and explain why not.*

Note that full information on the approval of the study protocol must also be provided in the manuscript.

Clinical data

Policy information about clinical studies

All manuscripts should comply with the ICMJE guidelines for publication of clinical research and a completed CONSORT checklist must be included with all submissions.

Clinical trial registration	*Provide the trial registration number from ClinicalTrials.gov or an equivalent agency.*
Study protocol	*Note where the full trial protocol can be accessed OR if not available, explain why.*
Data collection	*Describe the settings and locales of data collection, noting the time periods of recruitment and data collection.*
Outcomes	*Describe how you pre-defined primary and secondary outcome measures and how you assessed these measures.*

Dual use research of concern

Policy information about dual use research of concern

Hazards

Could the accidental, deliberate or reckless misuse of agents or technologies generated in the work, or the application of information presented in the manuscript, pose a threat to:

No	Yes	
☐	☐	Public health
☐	☐	National security
☐	☐	Crops and/or livestock
☐	☐	Ecosystems
☐	☐	Any other significant area

Experiments of concern

Does the work involve any of these experiments of concern:

No	Yes	
☐	☐	Demonstrate how to render a vaccine ineffective
☐	☐	Confer resistance to therapeutically useful antibiotics or antiviral agents
☐	☐	Enhance the virulence of a pathogen or render a nonpathogen virulent
☐	☐	Increase transmissibility of a pathogen
☐	☐	Alter the host range of a pathogen
☐	☐	Enable evasion of diagnostic/detection modalities
☐	☐	Enable the weaponization of a biological agent or toxin
☐	☐	Any other potentially harmful combination of experiments and agents

Plants

Seed stocks	*Report on the source of all seed stocks or other plant material used. If applicable, state the seed stock centre and catalogue number. If plant specimens were collected from the field, describe the collection location, date and sampling procedures.*
Novel plant genotypes	*Describe the methods by which all novel plant genotypes were produced. This includes those generated by transgenic approaches, gene editing, chemical/radiation-based mutagenesis and hybridization. For transgenic lines, describe the transformation method, the number of independent lines analyzed and the generation upon which experiments were performed. For gene-edited lines, describe the editor used, the endogenous sequence targeted for editing, the targeting guide RNA sequence (if applicable) and how the editor was applied.*
Authentication	*Describe any authentication procedures for each seed stock used or novel genotype generated. Describe any experiments used to assess the effect of a mutation and, where applicable, how potential secondary effects (e.g. second site T-DNA insertions, mosiacism, off-target gene editing) were examined.*

ChIP-seq

Data deposition

☐ Confirm that both raw and final processed data have been deposited in a public database such as GEO.

☐ Confirm that you have deposited or provided access to graph files (e.g. BED files) for the called peaks.

Data access links<br>*May remain private before publication.*	*For "Initial submission" or "Revised version" documents, provide reviewer access links. For your "Final submission" document, provide a link to the deposited data.*
Files in database submission	*Provide a list of all files available in the database submission.*
Genome browser session<br>(e.g. UCSC)	*Provide a link to an anonymized genome browser session for "Initial submission" and "Revised version" documents only, to enable peer review. Write "no longer applicable" for "Final submission" documents.*

Methodology

Replicates	*Describe the experimental replicates, specifying number, type and replicate agreement.*
Sequencing depth	*Describe the sequencing depth for each experiment, providing the total number of reads, uniquely mapped reads, length of reads and whether they were paired- or single-end.*
Antibodies	*Describe the antibodies used for the ChIP-seq experiments; as applicable, provide supplier name, catalog number, clone name, and lot number.*
Peak calling parameters	*Specify the command line program and parameters used for read mapping and peak calling, including the ChIP, control and index files used.*
Data quality	*Describe the methods used to ensure data quality in full detail, including how many peaks are at FDR 5% and above 5-fold enrichment.*
Software	*Describe the software used to collect and analyze the ChIP-seq data. For custom code that has been deposited into a community repository, provide accession details.*

Flow Cytometry

Plots

Confirm that:

- [ ] The axis labels state the marker and fluorochrome used (e.g. CD4-FITC).
- [ ] The axis scales are clearly visible. Include numbers along axes only for bottom left plot of group (a 'group' is an analysis of identical markers).
- [ ] All plots are contour plots with outliers or pseudocolor plots.
- [ ] A numerical value for number of cells or percentage (with statistics) is provided.

Methodology

Sample preparation	*Describe the sample preparation, detailing the biological source of the cells and any tissue processing steps used.*
Instrument	*Identify the instrument used for data collection, specifying make and model number.*
Software	*Describe the software used to collect and analyze the flow cytometry data. For custom code that has been deposited into a community repository, provide accession details.*
Cell population abundance	*Describe the abundance of the relevant cell populations within post-sort fractions, providing details on the purity of the samples and how it was determined.*
Gating strategy	*Describe the gating strategy used for all relevant experiments, specifying the preliminary FSC/SSC gates of the starting cell population, indicating where boundaries between "positive" and "negative" staining cell populations are defined.*

- [ ] Tick this box to confirm that a figure exemplifying the gating strategy is provided in the Supplementary Information.

Magnetic resonance imaging

Experimental design

Design type	Event-related design
Design specifications	192 task trials per subject per session (two task sessions per subject). A trial lasted 9.15 - 11.15 seconds, plus an inter-trial interval of 4-5.5 seconds.<br>The 192 trials were distributed over 8 blocks of approximately 5 minutes each.
Behavioral performance measures	For every choice between a risky and a safe prospect, we recorded which option the participant chose, as well as their response time.

Acquisition

Imaging type(s): Functional and strucctural

Field strength: 3T and 7T

Sequence & imaging parameters:

3T data
We acquired functional MRI data using the Philips Achieva 3T whole-body MR scanner equipped with a 32-channel MR head coil, located at the Laboratory for Social and Neural Systems Research (SNS-Lab) of the UZH Zurich Center for Neuroeconomics. We collected 8 runs of fMRI data with a T2*-weighted gradient-recalled echo-planar imaging (GR-EPI) sequence (150 volumes + 5 dummies; flip angle 90 degrees; TR = 2286 ms, TE = 30ms; matrix size 96 × 70, FOV 240 × 175mm; in-plane resolution of 2.5 mm; 39 slices with thickness of 2.5 mm and a slice gap of 0.5mm; SENSE acceleration in phase-encoding direction (left-right) with factor 1.5; time-of-acquisition 4:52 minutes). Additionally, we acquired high-resolution T1weighted 3D MPRAGE image (FOV: 256 × 256 × 170 mm; resolution 1 mm isotropic; Shot TR = 2800ms; TI = 1098.6 ms; 256 shots, flip angle 8 degrees; TR = 8.3 ms; TE = 3.9 ms; SENSE acceleration in left-right direction 2; time-of-acquisition 5:35 minutes).

7T:
We also acquired MRI data using a Philips Achieva 7T whole-body MR scanner (located at the Institute for Biomedical Technology of ETH Zurich) equipped with: 7T MRI quadrature transmit/32-channel head receive array coil (Nova Medical). For the functional data, we acquired T2*-weighed GRE-EPI sequences (150 volumes + 5 dummies); flip angle 74 degrees, TR=2300 ms; TE = 15ms; matrix size 109 x 128 (LR x PA); FOV 190 x 224 mm; in-plane resolution of 1.75 mm; 76 slices with thickness of 1.75 mm with no slice gap; SENSE-acceleration in phase-encoding direction (left-right) with factor 3; Multiband acceleration with factor 2; time-of-acquisition 5:35 minutes). At 7T, we acquired a T1-weighted 3D MPRAGE image with the following parameters: FOV = 240 × 240 × 160 mm; voxel resolution = 0.8 × 0.8 × 0.8 mm; inversion time (TI) = 1656.2 ms; 164 shots; flip angle = 7°; repetition time (TR) = 9.8 ms; echo time (TE) = 4.5 ms; SENSE acceleration factor = 3 (left-right) and 1.5 (anterior-posterior); total acquisition time = 4 minutes 43 seconds.

Area of acquisition: Whole-brain

Diffusion MRI: ☐ Used ☒ Not used

Preprocessing

Preprocessing software: We used fmriprep version 20.2.2

Normalization: All key analysis were performed in individual space. However, we used a mask of the right Intraparietal Sulcus based on Barretto-Garcia, 2023, which was defined in fsaverage space. To obtain the mask in individual (volumetric) space, we used standard freesurfer registration as implemented in fmriprep.

Normalization template: fsaverage

Noise and artifact removal: We used GLMSingle to estimate single-trial estimates, which uses a cross-validated version of compcorr. Thus, it extracts an optimized number of principal components out of voxels that show no clear task-related signal and uses them as a confound regressors in the single trial estimation.

Volume censoring: We applied no volume censoring.

Statistical modeling & inference

Model type and settings:

We used GLMSingle to estimate single trial BOLD responses.

These single-trial BOLD estimates were used as input to a numerical receptive field model, defined on the BOLD activity of individiual subjects. These numerical receptive fields were then inverted, to obtain single trial decoded uncertainties, which were then compared to behavioral responses on those trials.

Effect(s) tested: The decoded uncertainties were used for a median split for otherwise identical trials. They were also used as a linear regression into a psychometric model of the task.

Specify type of analysis: ☐ Whole brain ☒ ROI-based ☐ Both

Anatomical location(s): We used the numerical parietal cortex mask we constructed in Barretto-Garcia et al, 2023.

Statistic type for inference (See Eklund et al. 2016): We performed no voxel-wise or cluster-wise inference, only ROI-based analysis.

Correction: n/a

Models & analysis

n/a	Involved in the study
☐	☒ Functional and/or effective connectivity
☐	☐ Graph analysis
☐	☒ Multivariate modeling or predictive analysis

Functional and/or effective connectivity	*Report the measures of dependence used and the model details (e.g. Pearson correlation, partial correlation, mutual information).*
Graph analysis	*Report the dependent variable and connectivity measure, specifying weighted graph or binarized graph, subject- or group-level, and the global and/or node summaries used (e.g. clustering coefficient, efficiency, etc.).*
Multivariate modeling and predictive analysis	We used an inverted numerical receptive field (nPRF) model. We first fitted a voxelwise nPRF model that predicts single-trial BOLD responses to a given numerosity using Gaussian-shaped tuning. Then, we selected, in a cross-validated fashion, voxels that predicted out-of-sample BOLD responses with a explained variance more than zero. To those voxels, we fitted a multivariate t-distribution noise function. Together with the nPRFs of those voxels, that yielded a likelihood function that could be used to perform Bayesian decoding of presented numerosities based on out-of-sample BOLD data.<br>The decoded posteriors were also used to get an estimate of the uncertainty in the neural code on a particular trial.
