# Supplementary material for: Rapid changes in risk attitudes originate from Bayesian inference on parietal magnitude representations
Source: Nat Commun. 2026 Sep 10;17:10703. doi: 10.1038/s41467-026-77402-6 (PMC13658247; doi:10.1038/s41467-026-77402-6)
Supplement: Supplementary file 3 [file 41467_2026_77402_MOESM3_ESM.pdf]

nature portfolio

Peer Review File

Rapid Changes in Risk Attitudes Originate from Bayesian Inference on Parietal Magnitude Representations

Corresponding Author: Mr Gilles de Hollander

This file contains all reviewer reports in order by version, followed by all author rebuttals in order by version.

Version 0:

Reviewer comments:

Reviewer #1

(Remarks to the Author)
The paper brings an array of methods and datasources to demonstrate a relationship between numerical representation in the brain and risk preferences via a nuerocomputational model based on the principles of efficient coding. While previous work has demonstrated this result in behaviour at the subject-level, this paper demonstrates a trial-level relationship between numerosity (decoded from neural activity) and choice behaviour. The analysis is rigorous and the range of data sources linking neural variability, perception, and choice behaviour is impressive. Figure 4 is a highlight, particularly the effect on behaviour of decoded noise levels in neural signals and the implication for model parameters in 4D. In particular, on
on trials where neural representation of payoffs have larger estimated variance, the subjects choices are less consistent and more biased toward their prior. My issues with the paper lie in exposition (particularly int the introduction), the framing of the results, and the description of the study.

Major Issues

1) A paper which attempts to draw a link between numerical perception and choice behaviour over lotteries faces the issue of how to describe the choice behaviour. The standard terminology in economics for nearly a century has been to describe the willingness of a decision-maker to accept a sure thing lower than the expected value of a lottery as a “risk-averse preference”, that is, the DM has full information about the payoffs and probability (but not the true state underlying the lottery) and prefers to sacrifice some upside of the lottery in favour of a sure payoff. However a model in which the DM does not have full information about payoffs (i.e. a noisy coding model) instead explains some of this behaviour as the result of noisy coding and not to preference (i.e the subject can be risk neutral over the perceived lotteries but still choose the sure amount). While the standard nomenclature labels this a “preference,” under a noisy coding model it is not a preference, per se, instead the behaviour is due to noisy-coding of information. A great example is the recent paper by Netzer et al. (2024) in the JEEA, where they posit a risk neural agent, but then demonstrate how this agent will appear to have different risk attitudes due to noisy coding. At times, this paper use of the term “preference” is inconsistent with the neoclassical view, as well as with the view from the noisy-coding literature. I would suggest that the authors try following the conceptual framing and language in Netzer et al. Some examples include:

a) Pg1 “Classic choice theories explain such individual differences as varying preferences over larger and smaller gains…. according to this view, risk preferences reflect the extent to which a decision-maker….”. The use of the term “preference” here is not accurate. Preferences in neoclassical decision theory under risk are binary operators over the choice set (i.e an ordering). They are not defined over magnitudes of gains. And they are not behaviour (formally choices are defined as “revealed preferences” which is observable by the analyst). From Mas-Collel Def 6.C.1: An agent is defined to be risk averse if they prefer the expected value of a lottery to the lottery itself. The revealed preferences of the agent (i.e. choices) over a set of lotteries can then be examined. If behaviour satisfies the axioms of EU, then the behaviour can be rationalized by a utility function over magnitudes of gains with Diminishing Marginal Utility (which is what I assume the authors mean by “Characteristics of utility functions”). Different agents will make different decisions for the same lottery (i.e. choose the sure EV or lottery), therefore can be rationalized with different utility functions (i.e. curvature).

b) I see the main points of the argument as follows: Neo-classical choice theories explain individual differences as resulting from different preferences for risk: some agents will choose the sure expected value of a lottery over the lottery itself.

Different agents will make different decisions for the same lottery. Under EU, this behaviour can be rationalized with different utility functions (i.e. curvature). However, under noisy coding, "the representation does not reflect the DM's true preferences and welfare but rather the DM's errors in aggregating the sampled information" (Netzer et al., 2024).

c) Pg1: The second paragraph beginning with "Although some theorists…" is distracting from the main argument. The discussion of "attitudes" is unclear since these are not defined (and I don't understand the relation to "tractability", it seems unnecessary to the argument). How are attitudes mathematically tractable? Are the authors referring here to utility functions? It is true that working with utility functions is more tractable than working with preferences (binary operators) directly, but again, why does this matter for the argument? I don't understand why this third concept "attitudes" is being introduced. Isn't it just sufficient to say that 1) neo-classical theory holds that preferences are stable over the set of lotteries (this can be accompanied with the quote about the rocky mountains) 2) risky choice behaviour tends to differ across different contexts, 3) explaining this behaviour with preferences implies that preferences are not stable, and 4) this defeats the whole point of positing preferences in the first place?

d) "Accordingly most economic theories say very little about where risk preferences come from…" This is a feature of economic theory, not a bug. If the authors intend to say that "representation theorems do not say anything about where preferences come from", this is because this is the point of a representation theorem! A representation theorem like EU (or RDU or even Prospect theory) can put structure on decision problems without having to explain preferences. This is a convenient property for applications like prediction (as long as the axioms of the representation theorem are satisfied). They don't intend to explain preferences, they posit the existence of them, and then place axiomatic structure on them to explain/predict behaviour. Regardless, there have been some attempts to explain the source of risk preferences in economic theory, see Robson (2001) JEconLit, and Robson and Samluelson (2022) JEconTheory and citations within.

e) I would also note that the authors are not explaining where risk preferences come from either, rather they are explaining a source of variation in risky choice behaviour that is not due to preferences. My understanding of the author's perspective is that these should be different things. Therefore it is important to be consistent with terminology.

f) The citation to McFadden seems out of place, since those papers did not address choice over lotteries. The more appropriate cite is Gul and Pesendorfer (2006, Econometrica)

g) "Moreover, even when the exact same choice problem is presented repeatedly within the same context, participants still choose differently across repetitions." An alternative view (to noisy coding) on the source of this behaviour is reviewed by Agranov and Ortoleva, 2022.

h) "Notably, this agnostic stance… has changed in recent years." This framing of this paragraph contradicts what is written earlier (i.e 'economists have been only focused on preferences but have no explanation for them!'). I believe this literature should come earlier in the argument to justify the theoretical motivation of the paper. It is fine to say, in one paragraph, that the predominant view is preferences, some theoretical and empirical work has been done to show that it may be noisy coding, and we are going to demonstrate empirically (in both behaviour and neurons) that we can explain choices with noisy numerical representation. This is still a strong contribution.

i) "Risk-averse individuals might not actually have concave utility functions" First, utility functions are a representation that describe an individual's behaviour, so we don't need to get into a debate about whether subjects "have them". Second, do the authors mean that their subjects might be truly indifferent between a lottery and its expected value (if they could observe them perfectly)? Positing that there is no such thing as risk preference is a strong claim that is not clearly justified. Again, I suggest following the terminology in Netzer et al. 2024 here.

2) The discussion of the experimental paradigm is unclear and lacking some important details. The critical feature of the design is that the presentation order of the two choice alternatives (risky or safe) are randomized. This ensures that either risky or safe must be maintained in memory, and behaviour can be assessed from this manipulation. However the description emphasizes different features. For example,

a) "Crucially, the payoffs of the safe and risky options were presented sequentially as clouds of 1-Swiss Franc coins, preceded by a piechart and percentage information indicating whether the probability of a payoff was 55%or 100%". This is not the "crucial" feature of the design. This is describing the order of the presentation of rewards and probability within an option, which is held constant across the manipulation. It also contradicts the Figure 1 legend which states "Crucially, either the risky or the safe option was presented first and thus had to be held in working memory (all choice sets occurred twice for every order for every session)". Which is "crucial"? I believe it is the feature in the legend, not the one in the text.

b)Related to the sentence above, if the probabilities were presented first, and then the payoffs, why does the sentence reverse the order and describe payoffs before probabilities. This is just confusing. The description of the experiment needs to be re-written clearly

c) "Thus, options were presented either earlier or later in time, regardless of their riskiness." Is this just saying that the order of risky or safe was randomized? If so, then emphasize that this is the main manipulation of the study.

d) "However, note that we have recently shown that decisions based on symbolic (i.e., Arabic numerals) and non-symbolic (i.e., coin clouds) presentations of magnitudes are psychometrically very similar and rely on the

same approximate number system." Are the authors claiming that error rates and response times are similar between "is 500 larger 499" and assessing the size of a cloud of 500 vs 499 coins? This can't be true. This is a big jump that needs to defended in the intro or discussion rather than in the experimental description. The results section is not the place to defend this point. The limitation of the study on this point needs to be addressed.

e) In legend of Fig 1, a number of important features of the figures are not described. Instead, the legend draws conclusions that are not yet justified and need to be substantiated in the text itself:
"in line with that assumption that options presented first (last) are noisier (less noisy) and thus more prone to a central tendency effect." So far nothing can be concluded about the noisiness of the representation, only that behaviour is different. and "The probability that participants chose the risky option depended on the absolute magnitudes of the choice options, particularly when the risky option came first. This is a clear example of a preference reversal.

Why is this a clear example? So far all that has been noted is that noise (order) leads to more reliance on the prior. Why does that imply the subject is more likely to pick the last option presented (which is what 2B appears to tell us) or that this should lead to a preference reversal. What even the central tendency in this dataset that the perception is being biased toward? Is it the safe option?

f) The description of the experiment does not detail what is the distribution of payoffs over trials. Is it uniform? Were the middle stimuli (e.g. 10 14 20) more likely than the outliers (5 and 28). Relatedly, in 1C, why is blue u-shaped (i.e. why are 10 and 14 larger than 0.5)?

3)Though, the description of the model is reserved for the methods, the description in the main text and Figure 2A is missing some key features that make it difficult to understand the predictions. The description of the mechanisms in Figure 2A is not clear, there are a number of steps missing in the logic (in the legend). I would suggest breaking Figure 2A into its own separate figure where the key features of the model are described from the perspective of the experimental paradigm. This figure could also include the estimated densities given the model parameters (perhaps as a separate pane).

a) Figure 2A should be a representation of the model in the experimental paradigm. However it is not clear to me that it is. Why do the risky and safe options have different likelihoods in Safe First and Risky First Shouldn't they be the same (since safe and risky are randomized in order)?

b) Where, specifically, is the "Central tendency bias" on this diagram.

c)What is on the x axis for each of the these plots, EV? What is the posterior belief over?

d) My interpretation of the model is that it is essentially a semi-parametric application of KLW, where the parameters are allowed to differ across manipulations and there is not a common prior for both risky and safe. Are these the only differences? Be clear about the relative contribution here.

e) Given the estimated parameter values, why aren't the densities in 2A plotted using the actual estimated densities? Then the example would be relevant for explaining the data.
Why are there two plots for Risky-safe in Figure 2.D? And different scales of the x-axis?

Minor Issues

"This paves the way for the question whether there is acomparable relationship between neural noise and behaviour within single decision-makers." Kurtz-David et al. (2019) have studied this question in this journal, though not from the perspective of perceptual acuity. There does seem to be some relation between neural noise and behaviour at a trial-by-trial level not studying acuity, per se, however the source of this noise is not investigated.

Please put page/line numbers on the draft so that rewiewers can refer to them easily.

Figure 1. g) Is colour-coding in 1C (orange blue) still "risky first" and "safe first".
What is "Ratio Risky/Safe." This is not described in the figure legend.

"…the acuity with which different choice options are represented can dynamically change as a function of exogenous or endogenous context." Preseumably, exogenous context is manipulation of the order. What is "endogenous context" and how does this paper establish it? If it does not, then remove.

Figure 4 c. Where is risk neutrality in this figure? 0? The x-axis needs a label.

(Remarks on code availability)

Reviewer #2

(Remarks to the Author)
In this study, Hollander and colleagues use a combination of fMRI and computational modeling to argue that contextual

shifts and moment-to-moment fluctuations in risk preferences emerge from Bayesian inference on noisy magnitude representations in the parietal cortex. In their experiment, participants chose between a safe and a risky option, both represented as patches of dots (indicating reward magnitudes) with fixed probabilities (either 55% or 100%). In some trials, the safe option appeared first, while in others, the risky option appeared first, requiring participants to either hold the first option or second option in working memory.

They found that when risky options were held in working memory, participants were less likely to choose them (risk aversion) if they had large payoffs but more likely to choose them (risk-seeking) if they had small payoffs. The authors relate this preference reversal to change in risk preference and accounted for it using a computational model in which Bayesian inference on noisy magnitude estimates led to central tendency biases—causing small payoffs to be overestimated and large payoffs to be underestimated. Consistent with the behavioral modeling, fMRI results showed that trials with noisier intraparietal payoff representations were associated with less consistent and less risk-neutral choices.

Overall, the paper is well-written, and the study objectives are clear. However, as explained below, I do not believe this task actually measures changes in risk preference in the way it is typically defined in the field. The probabilities of the safe and risky options are fixed, and reward magnitudes are represented as patches of dots, making the task more akin to a perceptual decision-making task with reward manipulation. Since perceptual noise naturally plays a role in comparing the two options (because one option has to be held in WM), any resulting preference shifts between the two options are expected. However, this does not necessarily reflect changes in risk preference due to subjective value formation or differential weighting of reward probability and magnitude. As a result, I find the study's conclusions about risk preference unconvincing.

Major Concerns:

Problematic Task Design:
1) The fixed probabilities for the safe and risky options turn the task into a simple perceptual comparison between two dot patches rather than an actual value-based decision-making task involving risk.
2) The observed preference for the option presented last appears to be a perceptual bias rather than a reflection of risk attitudes. This can be seen in Figure 1, where the difference in selections for two conditions remains relatively the same for different dot ratios.
3) The task lacks variability in reward probabilities, which is essential for properly studying risk preferences.
4) Even if one argues that the task captures risk preferences due to the presence of a risky option, the perceptual noise in dot-based magnitude representation may not be relevant to the types of noise involved in traditional value-based decision-making tasks, where magnitudes are presented as explicit numbers. The authors should repeat their experiment using numerical reward values, which is often used in the field, and test whether an order effect still exists.

Limited Model Comparison:
5) The study compares the proposed model with only one alternative. A more basic model based on perceptual comparison of dot counts with varying precision could potentially explain the data just as well.

Trial-by-Trial Priors:
6) The authors attribute differences in choice behavior to priors (usual escape goat in Bayesian approach), but do not investigate whether priors are updated on a trial-by-trial basis. A model incorporating such adjustments would strengthen their claims.

Mechanistic Interpretation of a Prescriptive Model:
7) The authors describe their Bayesian model as "mechanistic," which is misleading. Bayesian models, by definition, are prescriptive (see Abbott, 2008). While the proposed model assumes different priors and noise levels, it does not explain *how* priors are shaped, *how* noise affects expected value estimation, or *how* decisions are made at the neural level. Bayesian models prescribe the way priors and noisy observations should be combined, but they do not necessarily reveal any mechanisms.

Missing but important results:
8) The authors do not report results from participants' uncertainty ratings, which could provide valuable insights. These ratings could reveal whether perceived certainty depends on the perceptual properties of the dot displays, whether order effects influence confidence, and whether uncertainty decoding from neural data aligns with subjective confidence reports.

(Remarks on code availability)

Version 1:

Reviewer comments:

Reviewer #1

(Remarks to the Author)
The authors have addressed my comments from the last round. The framing of the paper and its contribution is much-

improved. I want to emphasize that I think this is an excellent study that will make an exciting contribution to the literature on choice under uncertainty. While the existence of sequential presentation effects on risky choice are known in the literature, this paper provides both a rigorous theoretical mechanism as well as additional process data (numerical perception derived from fMRI of partial cortex) to justify the argument that variation in numerical perception is linked to choice behaviour. The array of methods and data sources are impressive and compelling. As noted in my last review, Figure 4 is a highlight and is an important contribution to our understanding of risky choice behaviour and how to measure risk preferences. While my comments with respect to framing may seem critical, they are intended to improve the paper so that it has maximum impact across a wide range of fields (including economic theory). I hope they are interpreted as an attempt to improve the paper for publication in Nature Communications. I have a few remaining comments, detailed below.

1) The addition of the behavioural task using Arabic numerals improves the paper and will help justify its conclusions for risky choice more broadly. I would suggest emphasizing this task, and the implication of its results, earlier in the introduction (lines 145-157). Currently this paragraph defends the use of the numerosity clouds to justify the fMRI experiment. All well and good. But a sentence here noting that the numeral task exists and that the behaviour was analyzed (and similar) across both tasks would help the reader place the following results in context. Currently, it's not until lines 236 that we learn that task even exists, and lines 570-590 that the behaviour is similar!

2) lines 84-90 In reference to Random Utility and recent advances in economic theory, the authors write:

"Although these theories alleviate the problem of choice inconsistency, they do not address the question of where this randomness comes from, or why risk attitudes are so dependent on context (e..g, order of presentation). Both theories frame the noise in observed behaviour as random and therefore external to the decision-making processes dealing with the choice-relevant information (e.g., payoffs). This stands in stark contrast to numerous findings in perceptual neuroscience that the degree of randomness in observed choices is usually tightly linked to the amount of bias in decisions, suggesting a common origin. In contrast to RUT, more recent economic choice models attribute risk attitudes directly to the noisiness of how decision-makers perceive the choice problems they are faced with."

This is not quite accurately framed. First, I agree with the author's main point that we need models in which the degree of randomness is linked to the amount of bias, and that the source of this randomness may be perceptual (or at least related to information acquisition). However it is not clear that this lies "In contrast to RUT…" rather these models may lie inside this class.

There are two theoretical issues here. First, to my knowledge it is not known whether an efficient coding model is, or is not, an RU model. It is important to note that the class of RU models is quite "large" in the sense that many other theories/models fall in this class. This is because the necessary and sufficient conditions for RU are rather weak (in terms of the restrictions they place on choice behaviour). A recent empirical example is given by McCausland et al. (2020). I believe the authors have in mind a specific version of a RU model in which stochastic utilities are assumed to be exogenous, independent of context, and from a narrow class of distributions. This model _does_ place stronger restrictions on behaviour. But that is only a subset of models in the class of RU. One simple example is violations of IIA, some of which RU can account for through the choice of error distribution. I am not aware of any theoretical demonstration that efficient coding models violate regularity (a necessary condition for RU). Rather, a more plausible conjecture is that efficient coding models fall inside the class of RU. If one interprets the encoding function m() as a utility functon(as Netzer et al. do), then efficient coding models relax the requirement that the utility function depends on context. This is most apparent in Payzan-LeNestour & Woodford (Fig 2) where they describe a simple efficient coding model on a single outcome dimension. The probability of an error is given by the difference in the means of the posterior (i.e. the difference in the utility function m()), just as in random utility. Except now the utility function and error distribution are jointly (optimally) chosen via the principles of efficient coding. This is the key contribution of efficient coding because the general class of random utility models do not necessarily impose this requirement (i.e. while not necessary, it is sufficient). Because the model described by LeNestour & Woodford falls under the class of random utility, it would not be accurate to say that efficient coding is not random utility. For the author's statement to be true, efficient coding models would need to violate the Block-Marshak inequalities (necessary and sufficient conditions for RU), and it is not obvious to me that they do. If the authors can justify this statement, then that is an important contribution in its own right. Note that the above argument provides sufficient motivation to study the author's question because we want to understand the efficient coding function, m(), how it depends on context, and what it implies for measuring risk preferences.

Second, there is another strain of theoretical literature which makes this point about random utility as well. Fosgerau et al. demonstrate an equivalence between Random Utility and Rational Inattention, such that any RU model has a RI interpretation. In a RI model, the noise in decisions arises from the agent optically selecting (or ignoring) information because of constraints/costs on information processing (Mackowiak et al.). See also Hebert and Woodford on dynamic information acquisition. Therefore the stochasticity in RU can be interpreted as arising from from optimal information acquisition and can depend on the context of the problem (i.e. which information is costly, and which payoffs are worth collecting information about). This falsifies the final sentence in the author's paragraph. Moreover, some of these findings are only a few years old, so would also qualify as "recent". To my knowledge, it is not known how RI models map on to efficient coding models (though the discussion above would suggest there is some overlap). Again, this is sufficient motivation to examine efficient coding models in the context of the author's study.

Minor Issues

What is "model stacking" — it is mentioned a few time throughout the paper but not described.

line 666 - "… evidently …" —do the authors mean "obviously" here instead?

References

Fosgerau, M., Melo, E., Palma, A. de & Shum, M. Discrete Choice and Rational Inattention: A General Equivalence Result. Int. Econ. Rev. 61, 1569–1589 (2020).

Hébert, B. & Woodford, M. Rational inattention when decisions take time. J. Econ. Theory 208, 105612 (2023).

Maćkowiak, B., Matějka, F. & Wiederholt, M. Rational Inattention: A Review. J. Econ. Lit. 61, 226–273 (2023).

McCausland, W. J., Davis-Stober, C., Marley, A., Park, S. & Brown, N. Testing the Random Utility Hypothesis Directly*. Econ. J. 130, 183–207 (2020).

Payzan-LeNestour, E. & Woodford, M. Outlier blindness: A neurobiological foundation for neglect of financial risk. J. Financial Econ. 143, 1316–1343 (2022).

(Remarks on code availability)

Reviewer #2

(Remarks to the Author)
I appreciate the authors' efforts to address my concerns and questions.

Regarding my main concern—namely, what the task actually measures—I disagree with the authors' claim that the task measures risk preference simply because it includes a risky option on each trial. Because of how reward magnitudes are presented and because reward probability of the risky option, which is supposed to introduce risk, is fixed across trials, the task does not meaningfully manipulate risk. Because probabilities are fixed, they can be absorbed into outcome evaluation, as suggested by Prospect Theory and other heuristic models of risky choice, reducing the task to a comparison of reward magnitudes. The issue is therefore not the gap between theoretical and observable manifestations of risk attitude, but rather the absence of any manipulation of risk (i.e., probabilities) in the first place. As implemented, the task is too simplistic to reveal anything about risk preference, which is why the findings and framing focus entirely on the representation of reward magnitude, with no real link to risk preference.

Changing reward probabilities is not strictly required to measure risk preference, but in the current experiment, with the probability of the risky option held constant, the task effectively reduces to comparing reward magnitudes in terms of expected outcomes. Moreover, as many have noted, risk aversion in Expected Utility Theory is not truly about risk in the sense of probability processing, but rather about how reward magnitude is processed. Prospect Theory is the framework that meaningfully addresses risk preference by incorporating probability distortion, which is entirely ignored here. In this context, it is unclear how one can propose a model of risk preference reversal when probability does not enter the model at all and everything depends on the representation of payoffs.
In another word, probability does not play a role in the observed behavior; the task involves comparisons of magnitudes. As such, the study is fundamentally about magnitude representation in a choice context involving a sure and a risky option, rather than about risk preference itself. One could replace the probability dimension with another dimension, such as effort (for example, choosing between two options that offer different reward magnitudes and differ by a fixed amount of effort), without changing the core results.

Regarding Figure 1C, my point was that within a given level of stakes, the probability of choosing the risky option is relatively similar across different payoff ratios. There is a small interaction effect for the "small stakes" condition, most likely because participants can count the dots, which largely eliminates the order effect. I am glad the authors agree that what they observe is a perceptual bias, but as I argue above, this does not link to risk preference. If reward magnitude is the only variable subjects process in this task, then any change in preference reflects changes in the representation of reward magnitude, not changes in risk preference. Naturally, a noisier representation of reward magnitude will directly translate into noisier behavior when there are no other factors that change across trials.

A more appropriate reference for different types of models is Dayan and Abbott (2001), while Abbott (2001) also addresses relevant points. That said, I agree with the authors that their proposed Bayesian model provides plausible mechanisms for the observed behavior, namely asymmetric processing of reward magnitude depending on order.

With respect to the new uncertainty-related analysis, I first thank the authors for including an analysis of the uncertainty reports. However, I am disappointed that the uncertainty reports do not reflect the order effect, which the authors attribute to noise in encoding that supposedly underlies risk preference. This result ultimately undermines their conclusions.

(Remarks on code availability)

Version 2:

Reviewer comments:

Reviewer #1

(Remarks to the Author)
Thank you to the authors for the latest revisions. I think this is an excellent paper with an impactful contribution to the literature on choice under uncertainty. My recommendation is to accept the manuscript.

With that said, I do I think it may be useful to clarify some confusion in the review process. I am not asking for any changes to be made to the manuscript unless the authors wish to include them, rather this point is simply made with the hope that it resolves further debate.

1) The textbook definition of risk aversion requires only the observation of a single choice of a single lottery. Below is Definition 6.C.1 in Mas-Collel, Whinston, and Green.

"A preference relation over lotteries is risk averse if, for any lottery L, the degenerate lottery that yields the expected value of L with probability one is weakly preferred to L itself."

Note the "for any lottery L" condition, it is not "for every" or for "multiple", only one lottery is needed. Therefore in the author's experiment, the behavioural observation of "risk aversion," as typically defined in economics, can be inferred from a choice between a single 50/50 lottery and a sure thing. Therefore the author's experiment is valid for measuring risk preferences as defined in economic theory.

The authors do not attempt to manipulate "risk" in their experiment. Rather, they demonstrate that the sequencing of lottery information can alter the lottery choice behaviour of subjects. This is the important insight of the paper!! By the definition of risk aversion noted above, this means that the standard behavioural observation used to identify "risk preference" is fraught, and that choice is therefore not sufficient to identify preferences. A similar theoretical point is made by Netzer et al., (2025) in which an agent with risk neutral preferences will behave in a manner that "appears risk averse". While previous studies have demonstrated manipulations which alter lottery choice behaviour, this study links the manipulation directly to numerical processing in the brain with a clear neuro-psychological mechanism. Therefore, as demonstrated by the authors, we should not infer risk preferences from a single 50/50 lottery due to noisy magnitude representation. This means that the field of economics needs to reconsider one of its key tenets: the link between behaviour and preferences. Assessing the predictions of Prospect Theory, and the subsequent "fourfold pattern," requires an experiment that manipulates probabilities. However the authors are not making claims about the broader collection of empirical results that are covered by Prospect Theory.

References

Mas-Collel, A., Whinston, M. D. & Green, J. Microeconomic Theory. (Oxford University Press, 1995).

Minor

Change "adding a random noise term to the utility function" to "assume that preferences are stochastic". The first phrase describes only a subset of RU models which assume an additive random component, this is not the full set of RU models as outlined in the last revision note.

(Remarks on code availability)

Reviewer #2

(Remarks to the Author)
Comment 1:

I am not persuaded by the authors' claim that their task measures risk attitude simply because one of the options is probabilistic, for the reasons I outlined above. In addition, the distinction they draw between "risk preference" and "risk attitude" is completely arbitrary. These terms are often used interchangeably, and when a difference is made, it is typically that "risk attitude" refers to more innate or stable tendencies, whereas "risk preference" emphasizes the more flexible, context-dependent aspects of risky decision-making (see, for example, Chapter 9 of the Neuroeconomics volume edited by Glimcher and Fehr).

In addition, although expected utility theory (EUT) "captures" risk aversion through a concave utility function, it does not truly explain risk preference, because it is not explicitly concerned with probability processing. The fact that some studies still rely on an outdated approach in which reward probability is not manipulated to study risk preference does not justify this practice. One can certainly observe the effects of risk preference using an option with probabilisitic outcome, but without systematically varying the key component of risk—reward probability—it is impossible to measure how probability processing contributes to behavior. This is why, in my view, the current study is effectively reduced to evaluating reward magnitude, with little or no connection to genuine risk preference.

Comment 2:

I completely disagree with the authors' interpretation and feel they are largely playing with terminology to support their claims. If the task manipulates only reward magnitude and keeps reward probability fixed, then it is not measuring risk preference in any meaningful sense, since risk preference—by standard definitions—concerns choices over uncertain outcomes that explicitly involve probability weighting and probability-sensitive tradeoffs.

Comment 3:
As I mentioned earlier, there is no disagreement between us regarding what the Bayesian model contributes.

Comment 4:
I maintain my position that the confidence results undermine the central claim of this study.

(Remarks on code availability)

Open Access This Peer Review File is licensed under a Creative Commons Attribution 4.0 International License, which permits use, sharing, adaptation, distribution and reproduction in any medium or format, as long as you give appropriate credit to the original author(s) and the source, provide a link to the Creative Commons license, and indicate if changes were made.
In cases where reviewers are anonymous, credit should be given to 'Anonymous Referee' and the source.
The images or other third party material in this Peer Review File are included in the article's Creative Commons license, unless indicated otherwise in a credit line to the material. If material is not included in the article's Creative Commons license and your intended use is not permitted by statutory regulation or exceeds the permitted use, you will need to obtain permission directly from the copyright holder.
To view a copy of this license, visit https://creativecommons.org/licenses/by/4.0/

Reviewer #1 (Remarks to the Author):

The paper brings an array of methods and datasources to demonstrate a relationship between numerical representation in the brain and risk preferences via a nuerocomputational model based on the principles of efficient coding. While previous work has demonstrated this result in behaviour at the subject-level, this paper demonstrates a trial-level relationship between numerosity (decoded from neural activity) and choice behaviour. The analysis is rigorous and the range of data sources linking neural variability, perception, and choice behaviour is impressive. Figure 4 is a highlight, particularly the effect on behaviour of decoded noise levels in neural signals and the implication for model parameters in 4D. In particular, on trials where neural representation of payoffs have larger estimated variance, the subjects choices are less consistent and more biased toward their prior. My issues with the paper lie in exposition (particularly in the introduction), the framing of the results, and the description of the study.

We thank the reviewer for the very positive evaluation and the helpful comments on the theoretical framing. We have implemented their suggestions and believe this has substantially improved the clarity of the paper.

Major Issues

1) A paper which attempts to draw a link between numerical perception and choice behaviour over lotteries faces the issue of how to describe the choice behaviour. The standard terminology in economics for nearly a century has been to describe the willingness of a decision-maker to accept a sure thing lower than the expected value of a lottery as a “risk-averse preference”, that is, the DM has full information about the payoffs and probability (but not the true state underlying the lottery) and prefers to sacrifice some upside of the lottery in favour of a sure payoff. However a model in which the DM does not have full information about payoffs (i.e. a noisy coding model) instead explains some of this behaviour as the result of noisy coding and not to preference (i.e the subject can be risk neutral over the perceived lotteries but still choose the sure amount). While the standard nomenclature labels this a “preference,” under a noisy coding model it is not a preference, per se, instead the behaviour is due to noisy-coding of information. A great example is the recent paper by Netzer et al. (2024) in the JEEA, where they posit a risk neural agent, but then demonstrate how this agent will appear to have different risk attitudes due to noisy coding. At times, this paper use of the term “preference” is inconsistent with the neoclassical view, as well as with the view from the noisy-coding literature. I would suggest that the authors try following the conceptual framing and language in Netzer et al. Some examples include:

We appreciate the reviewer's insightful comment regarding the inconsistencies in our nomenclature. We would like to briefly point out that, for better or for worse, in scientific disciplines outside of economics, particularly in the fields of neuroeconomics and judgement and decision making, the term "preference" is frequently used interchangeably with terms such as "elicited preference" or "(behavioral) risk attitude". In these fields, these terms are often directly equated with observed behavior (e.g. risky choice proportions). See for example the seminal paper by Huettel et al. (2006, Neuron), but also Chew et al. (PNAS, 2019), or the recent

large-scale studies by Mortazavi et al. (2025, PNAS nexus) and Pedroni et al. (2017, *Nature Human Behavior*). Having said that, we think that sticking to the nomenclature used by Netzer and colleagues can indeed improve the conceptual clarity of our paper. We have now substantially revised the original text along these lines (see below for more details).

a) Pg1 "Classic choice theories explain such individual differences as varying preferences over larger and smaller gains…. according to this view, risk preferences reflect the extent to which a decision-maker….". The use of the term "preference" here is not accurate. Preferences in neoclassical decision theory under risk are binary operators over the choice set (i.e an ordering). They are not defined over magnitudes of gains. And they are not behaviour (formally choices are defined as "revealed preferences" which is observable by the analyst). From Mas-Collel Def 6.C.1: An agent is defined to be risk averse if they prefer the expected value of a lottery to the lottery itself. The revealed preferences of the agent (i.e. choices) over a set of lotteries can then be examined. If behaviour satisfies the axioms of EU, then the behaviour can be rationalized by a utility function over magnitudes of gains with Diminishing Marginal Utility (which is what I assume the authors mean by "Characteristics of utility functions"). Different agents will make different decisions for the same lottery (i.e. choose the sure EV or lottery), therefore can be rationalized with different utility functions (i.e. curvature).

We agree with the reviewer that our use of the term "preference" was imprecise from the perspective of neoclassical economics. We are now more precise in our nomenclature and explicitly describe how we use this term [L57-80]:

> *People differ widely in their attitudes towards risk. In lab experiments, some people will forgo a lottery with a larger expected value for a substantially lower but certain payout, whereas others will choose the lottery1–9. Relatedly, in everyday life, some people are more willing than others to become self-employed, engage in new relationships, or to invest money in the stock market10. Neoclassical economic theories describe such individual differences in behavior using the concept of individual risk preferences: An individual would be risk-averse if they prefer to obtain for sure the expected value of a risky prospect (its gain multiplied by its probability) to realizing the risky prospect itself, risk-seeking if they prefer the risky prospect itself to obtaining its expected value for sure, and risk-neutral if they are indifferent between these options. These preferences are believed to be (relatively) stable and generalizable across choice domains3,4,11. Preferences are by definition latent and cannot be measured directly; however, they can be inferred from overt behavior (choices "reveal" preferences, in the terminology of neoclassical economics). Thus, measuring overt risk attitudes in laboratory choices is the standard way to infer underlying risk preferences, which is seen as key for any statements,*

recommendations, or interventions designed to help people take decisions under uncertainty in a way that increases their chances to meet personal goals.

Empirical data reveal, however, that individual risk attitudes are highly unstable12,13. Objectively identical choice sets (i.e., accept/reject specific gambles) can lead to different choices depending on the options that were offered before14 or in which order choice options are presented15. Furthermore, even when the exact same choice problem is presented repeatedly within the same context, decision-makers choose differently across repetitions7,16,17. This seems at odds with the idxea that these choices mainly reveal stable preferences.

b) I see the main points of the argument as follows: Neo-classical choice theories explain individual differences as resulting from different preferences for risk: some agents will choose the sure expected value of a lottery over the lottery itself. Different agents will make different decisions for the same lottery. Under EU, this behaviour can be rationalized with different utility functions (i.e. curvature). However, under noisy coding, “the representation does not reflect the DM’s true preferences and welfare but rather the DM’s errors in aggregating the sampled information“ (Netzer et al., 2024).

We thank the author for this concise summary of the logical argument. As the reviewer will notice, our revised introduction now follows this terminology and framing.

c) Pg1: The second paragraph beginning with “Although some theorists…” is distracting from the main argument. The discussion of “attitudes” is unclear since these are not defined (and I don’t understand the relation to “tractability”, it seems unnecessary to the argument). How are attitudes mathematically tractable? Are the authors referring here to utility functions? It is true that working with utility functions is more tractable than working with preferences (binary operators) directly, but again, why does this matter for the argument? I don’t understand why this third concept “attitudes” is being introduced. Isn’t it just sufficient to say that 1) neo-classical theory holds that preferences are stable over the set of lotteries (this can be accompanied with the quote about the rocky mountains) 2) risky choice behaviour tends to differ across different contexts, 3) explaining this behaviour with preferences implies that preferences are not stable, and 4) this defeats the whole point of positing preferences in the first place?

We agree with the reviewer that the distinction between 'risk attitudes' and 'risk preferences', as defined by Elke Weber in the cited paper, might not be helpful for framing our work, even more so now because it does not align with the nomenclature of Netzer et al. we employ. Therefore, we have left out this reference and distinction from the introduction and follow the reviewer’s recommendations concerning terminology and framing.

d) "Accordingly most economic theories say very little about where risk preferences come from…” This is a feature of economic theory, not a bug. If the authors intend to say that

“representation theorems do not say anything about where preferences come from”, this is because this is the point of a representation theorem! A representation theorem like EU (or RDU or even Prospect theory) can put structure on decision problems without having to explain preferences. This is a convenient property for applications like prediction (as long as the axioms of the representation theorem are satisfied). They don’t intend to explain preferences, they posit the existence of them, and then place axiomatic structure on them to explain/predict behaviour. Regardless, there have been some attempts to explain the source of risk preferences in economic theory, see Robson (2001) JEconLit, and Robson and Samluelson (2022) JEconTheory and citations within.

We agree with the reviewer that it might be unfair to critique EU and related theories for not explaining the origin of preferences, since this is besides the point of those theories. Therefore, we now refrain from this issue. However, we do point out that for economic theory to be conceptually and practically meaningful, preferences need to be relatively stable and need to be assessed in ways that truly reveal them rather than some other factors. This contrasts with many findings that risk attitudes, as measured in choice experiments and used as a putative proxy of risk preferences, are unstable and can reflect perceptual distortions, as we show in our manuscript.

e) I would also note that the authors are not explaining where risk preferences come from either, rather they are explaining a source of variation in risky choice behaviour that is not due to preferences. My understanding of the author’s perspective is that these should be different things. Therefore it is important to be consistent with terminology.

We agree with the reviewer that, working with a stricter definition of risk preferences, we do not explain where risk preferences come from either. Using the new, more consistent terminology of Netzer et al, we no longer make that claim but precisely state that the standard way of measuring risk preferences, via choices in experiments, reveals systematic perceptual biases that affect the interpretation of these measures in terms of risk preferences.

f) The citation to McFadden seems out of place, since those papers did not address choice over lotteries. The more appropriate cite is Gul and Pesendorfer (2006, Econometrica)

We thank the reviewer for this suggestion. We have updated the manuscript accordingly.

g) “Moreover, even when the exact same choice problem is presented repeatedly within the same context, participants still choose differently across repetitions.” An alternative view (to noisy coding) on the source of this behaviour is reviewed by Agranov and Ortoleva, 2022.

We thank the reviewer for pointing us to this interesting reference. We know briefly mention it in the revised introduction [L81-83]:

> *Adaptations of the preferential framework have tried to account for the stochasticity of risky choice by adding a random noise term to the utility function (Random Utility*

Theory, RUT)[18] or by assuming that decision-makers may have a preference for responding randomly[19].

h) "Notably, this agnostic stance… has changed in recent years." This framing of this paragraph contradicts what is written earlier (i.e 'economists have been only focused on preferences but have no explanation for them!'). I believe this literature should come earlier in the argument to justify the theoretical motivation of the paper. It is fine to say, in one paragraph, that the predominant view is preferences, some theoretical and empirical work has been done to show that it may be noisy coding, and we are going to demonstrate empirically (in both behaviour and neurons) that we can explain choices with noisy numerical representation. This is still a strong contribution.

We thank the reviewer for the suggestion. The resource-rational theories of risky choice are now introduced already in the 4th paragraph of the manuscript [L91-106]:

In contrast to RUT, more recent economic choice models attribute risk attitudes directly to the noisiness of how decision-makers perceive the choice problems they are faced with[27–31]. These perceptual models of risky choice draw on theories from perceptual neuroscience, framing decisions as fully rational solutions to optimization problems computed on inherently noisy representations of choice-relevant information. To make optimal choices, our brain makes the best of these noisy signals by applying principles of Bayesian inference[24,32] and/or efficient coding[26,33], which can lead to biases in percepts of the lottery payouts and probabilities. Note that, on average, such biases are beneficial because they reduce the average perceptual error, by employing prior information about the likely causes of sensory signals in case these are noisy and therefore unreliable. For example, there is a well-known positive correlation between the levels of noise in cognitive representations and central tendency (CT) bias[34]: With increased noise, participants tend to regress their percepts of stimulus values towards the mean value of earlier observed stimuli. The Bayesian interpretation of this is that, when noise increases, participants also increasingly revert to their prior beliefs about possible stimulus values – just as normative statistical theories prescribe[16,24].

i) "Risk-averse individuals might not actually have concave utility functions" First, utility functions are a representation that describe an individual's behaviour, so we don't need to get into a debate about whether subjects "have them". Second, do the authors mean that their subjects might be truly indifferent between a lottery and its expected value (if they could observe them perfectly)? Positing that there is no such thing as risk preference is a strong

claim that is not clearly justified. Again, I suggest following the terminology in Netzer et al. 2024 here.

We agree with the reviewer and have now rewritten this statement without referring to the existence or non-existence of utility functions, and sticking to the main point that perception can change behavioral risk attitudes [L107-113]:

> *In the context of risky choices, decision-makers with noisier perception of a choice problem therefore tend to estimate the larger (risky) payoffs downward towards a prior mean, but the smaller (safe) payoffs upwards. As a consequence, decision-makers may underestimate the size of the larger risky payoffs, thereby exhibiting more risk-averse attitudes. This suggests that two people might in fact be similarly risk-neutral, but may show substantially different risk attitudes in their choices simply because they differ in how precisely and thus unbiased they can perceive a risky choice problem.*

2) The discussion of the experimental paradigm is unclear and lacking some important details. The critical feature of the design is that the presentation order of the two choice alternatives (risky or safe) are randomized. This ensures that either risky or safe must be maintained in memory, and behaviour can be assessed from this manipulation. However the description emphasizes different features. For example,

a) “Crucially, the payoffs of the safe and risky options were presented sequentially as clouds of 1-Swiss Franc coins, preceded by a piechart and percentage information indicating whether the probability of a payoff was 55%or 100%”. This is not the “crucial” feature of the design. This is describing the order of the presentation of rewards and probability within an option, which is held constant across the manipulation. It also contradicts the Figure 1 legend which states “Crucially, either the risky or the safe option was presented first and thus had to be held in working memory (all choice sets occurred twice for every order for every session)”. Which is “crucial"? I believe it is the feature in the legend, not the one in the text.

We thank the reviewer for pointing out that the description of the paradigm in the results section was confusing. Indeed, the critical feature of the design is the fact that the options are presented in different orders. How exactly the riskiness of the options was indicated (with the piecharts) is less important to the experimental design. We have therefore now left it out of this sentence and point out just the key details of the paradigm - this should now be much easier to follow [L178-184]:

> *In our paradigm, participants had to choose between a sure payoff and a gamble with a 55% probability of winning a larger payoff (Fig. 1A). Crucially, the payoffs of the safe and risky options were presented sequentially; in some trials, the risky options were presented first, in other trials, the safe option was presented first. This*

design aimed to introduce working memory noise for the first-presented option that had to be memorized, thereby presumably enhancing CT biases for this option.

b)Related to the sentence above, if the probabilities were presented first, and then the payoffs, why does the sentence reverse the order and describe payoffs before probabilities. This is just confusing. The description of the experiment needs to be re-written clearly

See the response above. We have removed the description of how the probabilities precede the payoffs, since it distracts from the key design feature.

c) "Thus, options were presented either earlier or later in time, regardless of their riskiness." Is this just saying that the order of risky or safe was randomized? If so, then emphasize that this is the main manipulation of the study.

We have implemented this suggestion of the reviewer. See the response to a).

d) "However, note that we have recently shown that decisions based on symbolic (i.e., Arabic
numerals) and non-symbolic (i.e., coin clouds) presentations of magnitudes are psychometrically very similar and rely on the same approximate number system." Are the authors claiming that error rates and response times are similar between "is 500 larger 499" and assessing the size of a cloud of 500 vs 499 coins? This can't be true. This is a big jump that needs to defended in the intro or discussion rather than in the experimental description. The results section is not the place to defend this point. The limitation of the study on this point needs to be addressed.

We appreciate this remark of the reviewer, which often comes up when we discuss our results with other researchers, in particular with economists. Although we appreciate the skepticism of the reviewer, there is substantial evidence for strong correlations between the acuity with which people can perceive numerosity and how well they can make quick decisions about different types of magnitudes, including Arab numerals. Note that our previous study actually provides these correlations as well, see Barretto-García et al, 2023, Nature Human Behavior. Moreover, it is well known that when people are making quick decisions about symbolically presented numbers, one does observe number-distance RT and even accuracy effects (e.g., Moyer & Landauer, Nature, 1967). See also Walsh (2003) for a comprehensive review. We discuss this literature in the discussion of the original, submitted manuscript.

Having said that, we agree with the reviewer that the results section might not be the optimal place to make this point. We have now removed this statement from the results section and motivate our use of the particular stimuli in the introduction. Moreover, we elaborate on how Arab numerals also draw on the general approximate number system in the discussion section.

See introduction [L145-157]:

In the following, we will first describe the risky choice paradigm we developed to modulate the acuity with which choice options are represented in the brain, by manipulating the order in which they are presented. This experimental paradigm also allows us to extract an fMRI signal that corresponds specifically to the neural acuity of the perception of the payoff of the first choice option, independent from any choice-related neural activity that comes later in the trial[35]. To be able to do so, we used the numerical magnitudes of dot clouds to present the payoff magnitudes to the participants, as we have in earlier work[35]. Such stimuli are somewhat different from the Arab numerals often employed to present potential payoffs; however, seminal work in numerical cognition has established that the neural processing of the numerosity of such stimuli is similar to that of more general (symbolic) numerical information[37,38], including Arab numerals[35]. Numerosity processing for both types of stimuli engages a more general parietal 'magnitude system'[39], and the acuity of numerosity processing for both types of stimuli is correlated across individuals[35].

And in the discussion [L685 - 705]:

One might argue that this choice context may induce effects in a purely 'low-level perceptual' manner that should be absent for choices with more common Arabic numerals. However, there is ample evidence that the perception of Arabic numerals is prone to similar perceptual effects[67]. For example, when participants have to make decisions about rapidly-presented Arabic numerals, they are sensitive to order effects[66] and the underlying statistics of the presented numerals[74], just as for the non-symbolic presentation formats in the study presented here. Furthermore, recent neuroscience studies have shown that numerosity-tuned regions similarly represent both symbolic Arabic numerals and non-symbolic stimulus arrays[75]. In line with this, our recent work[35] has shown that the performance on a perceptual task involving the numerosity of stimulus arrays similarly predicts choice consistency and risk attitude in risky choice tasks that present payoffs either non-symbolically or as Arab numerals. Finally, in the present study, we also presented a choice task where the non-symbolic stimulus arrays were replaced with unambiguous, Arab numerals. In that experiment, we also found an effect of order on risk attitude, as well as an interaction with stake size. A closer look at the behavior and parameter estimates of individual participants showed a wide array of potential choice behavior that could

not be explained by a standard expected utility model (which was also confirmed with formal model comparison measures). All this highlights that choice problems presented in non-symbolic and symbolic presentation formats engage similar neural magnitude representations, and that our model can capture common processes underlying choices about payoffs presented in both presentation formats..

We now also include an additional experiment (detailed in the Results and further expanded in Supplementary Text 3), which mirrors the fMRI experiment but uses Arab numerals rather than non-symbolic dot clouds. Our findings demonstrate that presentation order continues to influence risk attitude even with Arab numerals. Critically, this order effect also interacts with stake size, somewhat more subtly, but paralleling the pattern observed with dot-cloud numerosities. These results confirm that Arab numerals, just like the non-symbolic stimuli, are subject to central tendency biases consistent with Bayesian perceptual mechanisms.

e) In legend of Fig 1, a number of important features of the figures are not described. Instead, the legend draws conclusions that are not yet justified and need to be substantiated in the text itself:
“in line with that assumption that options presented first (last) are noisier (less noisy) and thus more prone to a central tendency effect.” So far nothing can be concluded about the noisiness of the representation, only that behaviour is different.

and “The probability that participants chose the risky option depended on the absolute magnitudes of the choice options, particularly when the risky option came first. This is a clear example of a preference reversal.

Why is this a clear example? So far all that has been noted is that noise (order) leads to more reliance on the prior. Why does that imply the subject is more likely to pick the last option presented (which is what 2B appears to tell us) or that this should lead to a preference reversal. What even the central tendency in this dataset that the perception is being biased toward? Is it the safe option?

We apologize that the captions of Figure 1 were probably not clear to readers who are not yet familiar with the whole study. We decided to restrict the caption of Figure 1 to describing the empirical data patterns and do not give any theoretical interpretations anymore [L199-213]:

> ***B)*** *Average proportion of risky choices (y-axis) as a function of the magnitude of the safe option (x-axis) and presentation order (hue). Participants were either more or less likely to choose the safe option when the risky option was presented first, depending on whether the stake size (i.e., indexed by the magnitude of the safe option, around which the magnitude of the risky option was varied) was larger or smaller. Error bars correspond to*

the standard error of the mean over participants. ***C)*** *Psychophysical curves for different stake sizes (defined as the magnitude of the safe option), i.e., the probability of a risky choice as a function of the ratio between risky and safe payoffs (binned per participant based on their calibration data, i.e., vincentization). Insets show the estimated risk-neutral probability, a measure of average risk attitude (RNP<55% = risk-averse, RNP>55% = risk-seeking). For relatively small-stakes gambles, participants become (more) risk-seeking when the risky option is presented first. Conversely, for relatively large-stake gambles, they became more risk-averse. This is in line with larger central-tendency effects for remembered versus currently presented payoffs.*

f) The description of the experiment does not detail what is the distribution of payoffs over trials. Is it uniform? Were the middle stimuli (e.g. 10 14 20) more likely than the outliers (5 and 28).

We thank the reviewer for pointing this out. For the sake of brevity, we had left these details out of the results section (since they are extensively described in the methods section). However, we agree with the reviewer that these details are important for the reader at this stage and now mention them in the results [L224-229]:

The safe option consistently offered one of six possible payoffs, with equal probability but in random order: 5, 7, 10, 14, 20, or 28 CHF. The risky option was determined based on one of eight possible ratios relative to the safe option. These ratios were established through a calibration task conducted outside the scanner, ensuring that the choices presented to participants were non-trivial, as they were near the participants' individual indifference points (see Methods for details).

Relatedly, in 1C, why is blue u-shaped (i.e. why are 10 and 14 larger than 0.5)?

Although the spread of log-ratios between the risky and safe option was identical for all stake sizes and orders, participants indeed slightly more often chose the risky option when the safe option was 10 or 14. Note, however, that this is a very subtle, qualitative pattern in the data: The standard errors of 5, 7 and 10 are still overlapping, so that one cannot conclude that this effect is reliable. Having said that, we also find this bump around 10/14 in our recent TMS study using the same paradigm (https://www.biorxiv.org/content/10.1101/2025.01.13.632678v1). In that study, we develop a more flexible PMC model that relaxes the assumption of Weber's law to account for this bump. However, these issues are outside the scope of this paper: They are simply irrelevant for the observed fluctuations that are the core topic of this study. Thus, to not distract the readership from the topic of the present study, we left these observations and the model adaptation out of this manuscript.

3)Though, the description of the model is reserved for the methods, the description in the main text and Figure 2A is missing some key features that make it difficult to understand the predictions. The description of the mechanisms in Figure 2A is not clear, there are a number of steps missing in the logic (in the legend). I would suggest breaking Figure 2A into its own separate figure where the key features of the model are described from the perspective of the experimental paradigm. This figure could also include the estimated densities given the model parameters (perhaps as a separate pane).

We thank the reviewer for this suggestion. We have now moved the theoretical panel of Figure 2 (Fig 2A) to the (right) of Figure 1. We have also annotated the figure more extensively to make it easier to interpret for readers that are not familiar with these types of models.
For example, the figure now explicitly says that the likelihoods and priors are over payoffs; it explicitly labels the different stages of the inference process; and there is an explicit "Central tendency"-annotation for the arrows between the likelihoods and prior.

See below for more detailed responses.

a) Figure 2A should be a representation of the model in the experimental paradigm. However it is not clear to me that it is. Why do the risky and safe options have different likelihoods in Safe First and Risky First Shouldn't they be the same (since safe and risky are randomized in order)?

We are sorry for the misunderstanding. The likelihoods for the (objectively) same option should be different for the two presentation orders, since options that are presented first are noisier than options that are presented second. We now make this clearer in the legend by including an explicit example [L213-222].

> *Illustration of the intuition behind the Perception and Memory-based Choice (PMC) model: When an option is presented first, its representation will be noisier because the option needs to be held in working memory and the participant has no direct access to the corresponding stimulus during the decision (unlike the second option). Thus, the very same risky option has a noisier likelihood when presented first (left panel) as compared to when it is presented second (right panel).* ***The first option will therefore be more influenced by the prior than the second option and will have a larger CT bias. Presentation order can therefore impact on risk attitude in different ways, depending on stake size. Note that this illustration uses plausible but stereotyped parameter values for visualization purposes.***

(Note that this panel has now moved to Figure 1D)

b) Where, specifically, is the "Central tendency bias" on this diagram.

The arrows that are pointing inwards from the likelihood towards the posterior represent the central tendency that occurs due to Bayesian inference. We have now explicitly indicated that with a "central tendency"-label (which is larger for the larger CT of the first-presented option).

c)What is on the x axis for each of the these plots, EV? What is the posterior belief over?

The posterior belief is over the payoff magnitudes, as discussed in the fourth paragraph of the introduction [L102-106]

> *With increased noise, participants tend to regress their percepts of stimulus values towards the mean value of earlier observed stimuli. The Bayesian interpretation of this is that, when noise increases, participants also increasingly revert to their prior beliefs about possible stimulus values – just as normative statistical theories prescribe[16,24].*

as well as where the model is described in the results section [L245-251]:

> *Thus, taking a perceptual Bayesian perspective[16,24,27,35], we predicted that the options that were presented first should be more sensitive to the central tendency (CT) bias, resulting in overestimation of relatively small payoffs and underestimation of relatively large payoffs. As a consequence, when a relatively small risky payoff was presented first, participants should be more likely to choose it compared to when it was presented second. Conversely, when a relatively large risky option was presented first, it should be less likely to be chosen.*

We have now also explicitly labeled the likelihoods and priors in the figure as being likelihoods/priors over payoffs.

d) My interpretation of the model is that it is essentially a semi-parametric application of KLW, where the parameters are allowed to differ across manipulations and there is not a common prior for both risky and safe. Are these the only differences? Be clear about the relative contribution here.

We would not label our PMC model as 'semi-parametric' (but this might be a field-specific definition issue). We believe the model nests the original Khaw/Li/Woodford-model by relaxing certain assumptions and adding additional parameters. Please note that the KLW model is ultimately also an application of earlier psychophysical models of number cognition (van Oeffelen et al., 1982, *Perception and Psychophysics*; Izard et al. 2008, *Cognition*) to economic choice problems. Our extension of these existing models, by relaxing certain assumptions about which parameters are necessarily fixed, allows us to describe new empirical patterns that

can be highly relevant for real-life economic decision making. As discussed in many papers on the relationship between gaze and economic choice (and in the discussion of our manuscript), the order in which people consider different options can have a profound impact on choice. Our model helps us microfound those empirical patterns in normative computational models of numerosity perception. We have now made this more explicit in the results section as follows [L275-282]:

> *The model builds on classic psychophysical models of number perception[49,50] as well as on economic models incorporating these insights to describe risky choice as a perceptual inference process, showing that risk aversion can arise as a result of central tendency effects in perception of the potential payoffs[27,35]. The crucial new feature compared to earlier models is that the acuity with which the payoffs of different choice options are represented can dynamically change as a function of exogenous (i.e., choice architecture) or endogenous (i.e., neural/physiological state) context.*

e) Given the estimated parameter values, why aren't the densities in 2A plotted using the actual estimated densities? Then the example would be relevant for explaining the data.

We chose an illustration that used stereotyped parameter values to point out the general mechanisms of the model. The 'actual estimated densities' are distributions at the level of the group parameters, not at the level of a single trial as used to depict the principle of the model. For every single trial and every single participant, these values can be (very) different, also because the payoffs are different on every trial. Arbitrarily choosing any parameter value from these posterior distributions would in our eyes be less helpful than the present schematic, for illustrating the general mechanisms of the modelthat explain the qualitative effects found in the empirical data.

(Note that we have moved this figure now to Figure 1D, as suggested by R1)

We now explicitly mention that these parameter values are handpicked for the purpose of illustration [L221-222]:

> *Note that this illustration uses plausible but stereotyped parameter values for visualization purposes.*

Why are there two plots for Risky-safe in Figure 2.D? And different scales of the x-axis?

We apologise for our oversight here, these panels should have included clearer annotations. The figure (now Fig. 2B/C) now contains explicit labels describing that these are the individual difference posterior estimates for the evidence sd (leftmost panel) between the 1st and second option, and the prior mean (middle panel) and prior std (rightmost panel).

Minor Issues

“This paves the way for the question whether there is a comparable relationship between neural noise and behaviour within single decision-makers.” Kurtz-David et al. (2019) have studied this question in this journal, though not from the perspective of perceptual acuity. There does seem to be some relation between neural noise and behaviour at a trial-by-trial level not studying acuity, per se, however the source of this noise is not investigated.

We thank the reviewer for this reference and we now briefly refer to it [L418-421]:

> *This paves the way for the question whether there is a comparable relationship between neural noise of choice problems representations and behaviour within single decision-makers (in addition to neural noise in putative downstream value computations[59]).*

Please put page/line numbers on the draft so that rewiewers can refer to them easily.

Thanks, we have now done so.

Figure 1. g) Is colour-coding in 1C (orange blue) still “risky first” and “safe first”.
What is “Ratio Risky/Safe.” This is not described in the figure legend.

We apologise for the oversight. We have strived to always use the same colors for certain data subsets (e.g., presentation orders), but to make this even clearer, we have now included the legend in both panels.

We now also include the ratio risky/safe in the legend [L205-213]:

> ***C)** Psychophysical curves for different stake sizes (defined as the magnitude of the safe option), i.e., **the probability of a risky choice as a function of the ratio between risky and safe payoffs** (binned per participant based on their calibration data, i.e., vincentization). Insets show the estimated risk-neutral probability, a measure of average risk attitude (RNP<55% = risk-averse, RNP>55% = risk-seeking). For relatively small-stakes gambles, participants become (more) risk-seeking when the risky option is presented first. Conversely, for relatively large-stake gambles, they became more risk-averse. This is in line with larger central-tendency effects for remembered versus currently presented payoffs.*

“…the acuity with which different choice options are represented can dynamically change as a function of exogenous or endogenous context.” Preseumably, exogenous context is

manipulation of the order. What is “endogenous context” and how does this paper establish it? If it does not, then remove.

Endogenous context means the current (physiological) brain state. This is now explained in the paragraph that comes after this sentence [L279-287]:

> *The crucial new feature compared to earlier models is that the acuity with which the payoffs of different choice options are represented can dynamically change as a function of exogenous (i.e., choice architecture) or endogenous (i.e., neural/physiological state) context. For example, in our particular experiment, the perception of the first- and second-presented payoffs can be differentially precise, as the noisiness of the neurocognitive payoff representation kept in working memory can be expected to be higher than the representation of the currently perceived payoff[46]. Furthermore, we can also use physiological indices of brain state (e.g., an fMRI activity decoding algorithm) to study how endogenous noise impacts choice behavior.*

Figure 4 c. Where is risk neutrality in this figure? 0? The x-axis needs a label.

Risk-neutrality is where the distance to risk-neutrality is zero. We now explicitly mention this in the legend to make this clearer [L540-543]:

> ***Distribution of plausible values of the individual differences between risk-neutral behavior (RNP = 55%) and their actual indifference points. Thus, a distance of 0 would correspond to perfectly risk-neutral behavior.***

We would again like to thank this reviewer for the very positive evaluation of our work and the many insightful tips on how to clarify the framing of our study and the presentation of our results.

Reviewer #2 (Remarks to the Author):

In this study, Hollander and colleagues use a combination of fMRI and computational modeling to argue that contextual shifts and moment-to-moment fluctuations in risk preferences emerge from Bayesian inference on noisy magnitude representations in the parietal cortex. In their experiment, participants chose between a safe and a risky option, both represented as patches of dots (indicating reward magnitudes) with fixed probabilities (either 55% or 100%). In some trials, the safe option appeared first, while in others, the risky option appeared first, requiring participants to either hold the first option or second option in working memory.

They found that when risky options were held in working memory, participants were less likely to choose them (risk aversion) if they had large payoffs but more likely to choose them (risk-seeking) if they had small payoffs. The authors relate this preference reversal to change in risk preference and accounted for it using a computational model in which Bayesian inference on noisy magnitude estimates led to central tendency biases—causing small payoffs to be overestimated and large payoffs to be underestimated. Consistent with the behavioral modeling, fMRI results showed that trials with noisier intraparietal payoff representations were associated with less consistent and less risk-neutral choices.

Overall, the paper is well-written, and the study objectives are clear. However, as explained below, I do not believe this task actually measures changes in risk preference in the way it is typically defined in the field. The probabilities of the safe and risky options are fixed, and reward magnitudes are represented as patches of dots, making the task more akin to a perceptual decision-making task with reward manipulation. Since perceptual noise naturally plays a role in comparing the two options (because one option has to be held in WM), any resulting preference shifts between the two options are expected. However, this does not necessarily reflect changes in risk preference due to subjective value formation or differential weighting of reward probability and magnitude. As a result, I find the study's conclusions about risk preference unconvincing.

We thank the reviewer for their kind words and for sharing their very relevant perspective on what our task can and cannot measure. We agree that our task does not allow us to measure changes in risk preferences due to subjective value formation or differential weighting of reward probability and magnitude. However, we believe this critique somewhat misses the central point of our study.

Ultimately, all that an experimenter can measure, *in any task,* is overt behavior—that is, *risk attitudes*. Risk preferences can by definition not be measured (see also the remarks of R1). As has been clearly demonstrated in a plethora of prior work, choice behavior (that is: risk attitudes) is profoundly influenced by how choice problems are presented and perceived. Therefore, we argue it is of utmost importance—both for economics and adjacent fields—to better understand how perception influences overt risk attitudes. Note that multiple recent, well-discussed studies argue along similar lines (e.g., Woodford, Netzer, Vieider, Frydman, Oprea).

By investigating how perception underlies observed risk attitudes, we can thus get closer to achieving two crucial goals: (a) estimating 'true' underlying risk preferences

by accounting better for perceptual and representational distortions, and (b) explaining real-world decision-making in environments with different choice architectures, which, for example, make specific attributes of choices more or less salient by presenting them earlier/later.

We suspect that some of the misunderstandings of our original manuscript may stem from differing definitions of what it means to study risk preferences. Our aim is to understand how the neurocognitive representation of choice options shapes the behavior that is typically interpreted as a direct proxy to such preferences.

To mitigate any possible misunderstanding, and in alignment with Reviewer 1's comments, we have substantially revised the introduction to more clearly distinguish between *risk preferences*—as a latent, theoretical construct—and *risk attitudes*—as the observable manifestations of those preferences under specific perceptual and cognitive conditions. We hope this makes our theoretical position and the contribution of our manuscript more transparent.

See Introduction [L54 - 76]

> *Different people have different attitudes towards risk. In lab experiments, some people will forgo a lottery with a larger expected value for a substantially lower but certain payout, whereas others will choose the lottery[1–9]. Similarly, in everyday life, some people are more willing than others to become self-employed, engage in relationships, or to invest money in the stock market[10]. Neoclassical economic theories describe such individual differences in behavior using the concept of individual risk preferences: An individual would be **risk-averse** if they prefer to obtain for sure the expected value of a risky prospect (its gain multiplied by its probability) to realizing the risky prospect itself, **risk-seeking** if they prefer the risk prospect itself to obtaining its expected value for sure, and **risk-neutral** if they are indifferent between these options. These preferences are believed to be (relatively) stable and generalizable across choice domains[3,4,11]. Preferences are by definition latent and cannot be measured directly. However, they can be inferred from overt behavior (choices "reveal" preferences, in the terminology of neoclassical economics). Thus, measuring overt risk attitudes is the standard way to infer underlying risk preferences, which is seen as key for any statements, recommendations, or interventions designed to help people take decisions under uncertainty in a way that increases their chances to meet their personal goals.*
>
> *Empirical data reveal, however, that individual risk attitudes are highly unstable[12,13]. Objectively identical choice sets (i.e., accept/reject a specific gamble) can lead to different choices depending on the options that were offered before[14] or in which*

order choice options are presented[15]. Furthermore, even when the exact same choice problem is presented repeatedly within the same context, decision-makers choose differently across repetitions[7,16,17]. This seems at odds with the idea of stable preferences being revealed by these choices.

Major Concerns:

Problematic Task Design:
1) The fixed probabilities for the safe and risky options turn the task into a simple perceptual comparison between two dot patches rather than an actual value-based decision-making task involving risk.

We thank the reviewer for their perspective, but note that their definition of a task measuring choices under risk as having to comprise changing probabilities across trials is clearly at odds with textbook definitions of risk aversion, as well as the existing empirical literature. As pointed out by Reviewer 1, the classic definition of risk aversion is as follows: "An agent is defined to be risk averse if they prefer the expected value of a lottery to the lottery itself." (Mas-Collel Def 6.C.1). This is exactly the type of choice that our participants had to make: Either a fixed amount of money, or a lottery with the same/similar expected value, but a larger variance of outcomes. This also follows from standard expected utility theory – widely considered as a first approximation to risky choice behavior and the fundament on which prospect theory is built. EU theory contains no probability distortions and therefore does not require varying probabilities across trials to be interpretable. Of course, we do not deny that probability distortions exist, but they are not the focus of our study.

Note that many recent as well as seminal papers on risky decision-making have used similar designs like ours (e.g. Nosteller-Mogee, 1951, Khaw, Li Woodford, 2021; Baretto-Garcia et al., 2023). Designs involving only accepting/rejecting 50-50% gambles with fixed probabilities are even more common in the literature (Tom et al., 2007, Science, de Martino et al., 2007, PNAS).

Of course, the question of how perceptual/valuation distortions of payoffs interact with varying probabilities is interesting and relevant, and researchers are starting to approach such questions using perceptual models as well (e.g., Khaw, Li, Woodford 2022, NBER, Vieider et al., 2024, Management Science). However, this issue is beyond the scope of the present study.

While we thus agree that designs with varying probabilities can be interesting and helpful to study perceptual probability distortions, there are equally good reasons for choosing to keep probabilities fixed. In particular, for the study in question, this helps to keep the computational modeling tractable and to optimize the number of trials that we could fit our neurocomputational model to. We now explain this design aspect in more detail, to justify our design choice and avoid misunderstandings [L185-190]:

We kept the payout probability of the risky option fixed at 55%, in line with previous studies[27,35], to streamline the computational modeling of behavioral and neural data. While we recognize the significance of probability distortion in decision-making[42], our study concentrated on the perceptual impacts on payoff evaluations. This focus required a design that emphasized statistical power for these specific effects, hence the fixed payoff probabilities.

2) The observed preference for the option presented last appears to be a perceptual bias rather than a reflection of risk attitudes. This can be seen in Figure 1, where the difference in selections for two conditions remains relatively the same for different dot ratios.

We apologize if this was not clear, but Figure 1C clearly shows an interaction effect between stake size and order, as reported in the text ("but crucially also the predicted interaction effect between order and magnitude on risk attitude. Moreover, the observed effect is not symmetrical for risky and safe options (see also Figure 2 and 3) which indicates that participants are processing risky and safe options in a categorically different manner. This is also what follows from our extensive model comparison analyses, as discussed in the results section.
We fully agree with the reviewer that this effect is a 'perceptual bias' - but one that differs between risky and safe options and profoundly affects measured risk attitudes (even when using Arab numerals to present the payoffs, see below). This is actually the main point of our paper: Perceptual effects and the neurocognitive representation of the choice problem impact economic choices in ways that systematically affect the measurement of risk preferences.

3) The task lacks variability in reward probabilities, which is essential for properly studying risk preferences.

We respectfully disagree. See our response to point 1, the many studies in the literature that study risk preferences without varying probabilities, and also the textbook definition given by referee 1 that does not contain any reference to the need to vary probabilities in order to study risk preferences.

4) Even if one argues that the task captures risk preferences due to the presence of a risky option, the perceptual noise in dot-based magnitude representation may not be relevant to the types of noise involved in traditional value-based decision-making tasks, where magnitudes are presented as explicit numbers. The authors should repeat their experiment using numerical reward values, which is often used in the field, and test whether an order effect still exists.

We thank the reviewer for this suggestion. We have now performed this experiment using Arabic numerals for the payoff representation in 58 subjects. It is concisely discussed in the results and more comprehensively described in Supplementary Text

3. In brief, there is a very strong order effect and also an interaction with stake sizes, just as in the fMRI experiment.

This confirms our thesis that perceptual/memory noise not only affects choices based on non-symbolic stimuli, but also decisions based on Arab numerals. Note that this has also been shown in earlier work where participants had to integrate over sequentially presented numerical information involving Arab numerals, both in a perceptual (Prat-Carrabin & Woordford, 2022, Nature Human Behavior; Tsetsos et al., 2012, PNAS) and risky choice context (Molter & Mohr, 2021, OSF).

Importantly, the conclusion that non-symbolic and symbolic magnitude information is processed in a similar way also aligns with our earlier work, which showed strong correlations in the consistency and bias with which people make perceptual decisions as well as risky choices presented as dot clouds and symbolic, Arab numerals (García-Barretto, 2023, Nature Human Behavior). We have now emphasized this point also in the introduction of the paper [L147-155]:

> *This experimental paradigm also allows us to extract an fMRI signal that corresponds specifically to the neural acuity of the perception of the payoff of the first choice option, independent from any choice-related neural activity that comes later in the trial[35]. To be able to do so, we used the numerical magnitudes of dot clouds to present the payoff magnitudes to the participants, as we have in earlier work[35]. Such stimuli are somewhat different from the Arab numerals often employed to present potential payoffs; however, seminal work in numerical cognition has established that the neural processing of the numerosity of such stimuli is similar to that of more general (symbolic) numerical information[37,38], including Arab numerals[35]. Numerosity processing for both types of stimuli engages a more general parietal 'magnitude system'[39], and the acuity of numerosity processing for both types of stimuli is correlated across individuals[35].*

Limited Model Comparison:
5) The study compares the proposed model with only one alternative. A more basic model based on perceptual comparison of dot counts with varying precision could potentially explain the data just as well.

We apologise if this was not clear, but our model does involve a 'perceptual comparison of dot counts with varying precision'. In its simplest form, "Model A" corresponds to the model by Khaw, Li, and Woodford and can be reparameterised to a standard probit psychophysical model, where

$$p(choose\ risky) = \Phi(\beta_0 + \beta_1 \log \frac{risky\ payoff}{safe\ payoff})$$

Critically, this model completely fails to account for the order effect and its interaction with stake, as evident in the qualitative model predictions (Figure 3A) as well as the formal model comparison (Figure 3E).

To make this clearer, we now added the following sentence to the model comparison section [L361-366]:

> *To do so, we compared the PMCM to four simpler models. The first model was a baseline model where the noisiness of the first and second options were identical and the priors were the same for risky and safe options (model A[27,35]).* ***Note that this model A is exactly equivalent to a standard probit psychophysical model, which models the precision with which decision makers can compare two numerosities (with a potential bias towards one of the two options).***

Trial-by-Trial Priors:
6) The authors attribute differences in choice behavior to priors (usual escape goat in Bayesian approach), but do not investigate whether priors are updated on a trial-by-trial basis. A model incorporating such adjustments would strengthen their claims.

We thank the reviewer for this interesting suggestion. The trial-to-trial adaptation is an idea we have considered for a few years. The problem with choice data is that there is very little information present in single trials. Thus, in our experience, it is very hard to fit such a model to these choice data. However, we are currently working on experimental data where participants had to indicate their "willingness to pay" or simply how many dots were in a stimulus array. Such data would be more suitable to ask these kinds of questions, but are not the scope of our present study.

We regret that the reviewer thinks the priors in our model are an "escape goat", but we respectfully disagree with this notion. Our data show clear effects of order and stake size that are fully in line with a Bayesian inference account involving priors, and that cannot be explained by any of the classic models employed in the field (the formal model comparison shows that a standard EU model or a standard probit psychophysical model cannot account for these effects). We cannot think of any other model that can plausibly explain these effects, but would be happy to perform corresponding model comparisons if the reviewer has a suggestion for other models that could explain these effects.

We would like to point out that currently we are collecting data sets where the objective prior of different options is explicitly manipulated to see if participants behave according to the model predictions (short answer: Yes). Note that other researchers have also done so (e.g., Frydman & Jing, 2021, Walasek et al., 2015) and find empirical effects that completely align with Bayesian inference and dynamic priors.

We now discuss these issues at in the discussion [L765-783]:

A central mechanism in Bayesian theories of (economic) cognition are prior beliefs about the state of the world. Some have argued that this may make Bayesian models of cognition overly flexible[97] (but see[98]). In the PMCM, decision-makers have prior beliefs over the plausible ranges of risky and safe payoffs. Because the mean and spread of these priors are free parameters, the PMCM is indeed a relatively flexible model. However, both formal model comparison and careful qualitative inspection of the empirical data show that less flexible models of risky choice (both of perceptual and preferential nature) can simply not account for the qualitative behavioral patterns in our data. Having said that, future work should validate whether change in the objective distributions of payoffs will result in the predicted behavioral changes and corresponding parameter shifts of the PMCM. Encouragingly, earlier work on both risk[30] and loss aversion[14] has already shown that human decision-makers are sensitive to the statistical properties of earlier choice problems, in line with Bayesian theories of economic choice. An important, related question is how, and on which time scale, human decision-makers can learn and adapt their subjective priors. After some initial attempts at including such a learning mechanism in our model, we believe that the choice paradigm we employed here is not suitable for such questions, because the amount of information present in a single accept/reject decision is limited. Therefore, future work might employ different paradigms with a more continuous behavioral measure, such as willingness-to-pay[99].

Mechanistic Interpretation of a Prescriptive Model:
7) The authors describe their Bayesian model as “mechanistic,” which is misleading. Bayesian models, by definition, are prescriptive (see Abbott, 2008). While the proposed model assumes different priors and noise levels, it does not explain *how* priors are shaped, *how* noise affects expected value estimation, or *how* decisions are made at the neural level. Bayesian models prescribe the way priors and noisy observations should be combined, but they do not necessarily reveal any mechanisms.

We thank the reviewer for their opinion. On the one hand, we agree with the reviewer that Bayesian models live at the 'computational level' of description (Marr, 1982) and of course do not explain at the ‘implementation level’ how such computations are instantiated by neural firing patterns in the brain (but see the work by Beck, Ma and Pouget from the 2000s). We are not sure why the reviewer cites Abbott 2008, because neither the word 'mechanistic' nor 'prescriptive' occurs in that paper, and because it does cite the seminal paper by Ma et al., in which "proposals have been made for how such computations might be performed at the neuronal level (Ma et al., 2006).". It is

exactly those (mechanistic) proposals that underlie our neurocomputational modeling and that drive home the point that Bayesian inference can actually be implemented in a neural system. We thus maintain that Bayesian models can be useful to understand the inner workings and outputs of such a neural system, even if they do not contain details of the neural processes implementing all inference steps.

Moreover, we note that our model clearly goes beyond the classic models of risky choice, which 'describe' choices using 'unobservable' utility functions. Crucially, models - that do not incorporate latent processes approximating cognitive functions that guide behavior cannot make predictions about how participants will choose in a new, unseen context. In contrast, our PMC model (as well as related models by e.g., Vieider and Woodford and colleagues) incorporate approximations of cognitive processes for which we have mechanistic insight (at the cognitive level) how these change with many contexts. The model can thus make precise predictions about how changing the noisiness of payoff stimuli, the stake sizes, or the current context change decision-making towards risk-aversion or risk-seeking. In addition, the model not only makes strong predictions about behavior, but also about neural signals in parietal cortex, which we then validate empirically. Both the measures of cognitive and the neural processes validate our model and explain individual and intra-individual choice variability. It is this consistency with cognitive and neural mechanisms, and their context-dependence, that in our eyes makes it appropriate to refer to our model as "mechanistic".

In sum, we agree with the reviewer that our model is not a full physical model of the neural computations that implement economic choice, but it clearly goes beyond current models of choices under uncertainty, by making strong and sometimes counterintuitive empirical predictions about both behavior and neural activity, which we experimentally validate. This is what we refer to as "mechanistic insight", not any claim that the model explains all aspects of neural function by which Bayesian inference is implemented in the brain. We now clarify this important point in the discussion and thank the reviewer for raising this important issue [L784-800]:

> *A related critique of Bayesian theories, and potentially the PMCM, is that they are not mechanistic[97] models of neural function but rather prescriptive models of information processing. Nevertheless, the PMCM does give us an increased understanding of the mechanism by which risk attitudes arise, through the lens of Bayesian perceptual inference and its link to neural activity. Concretely, without an understanding of working memory noise and the formalisation of its effects using the Bayesian framework, it remains very challenging to explain the rather complex observed stake x order interaction. Moreover, our neurocomputational modeling results link the abstract Bayesian formalism to concrete, neural signals that we observe using neuroimaging: Payoff magnitudes are represented in a non-linearly tuned population code in parietal cortex, which correlates with a posterior belief. The*

less consistent this population code across its subpopulations, the more uncertain the decision-maker's beliefs should be[32] and the more they should revert to their prior beliefs. Exactly this link between neural and behavioral signals, which forms a concrete decision-making mechanism, is what we observed in our data. While much work remains, our findings show how abstract, formal Bayesian theories of cognition can be integrated with measurements of corresponding neural population codes, which offers an interesting approach for gaining a more mechanistic understanding of decision-making.

Missing but important results:
8) The authors do not report results from participants' uncertainty ratings, which could provide valuable insights. These ratings could reveal whether perceived certainty depends on the perceptual properties of the dot displays, whether order effects influence confidence, and whether uncertainty decoding from neural data aligns with subjective confidence reports.

We agree with the reviewer that the uncertainty ratings are potentially very interesting, also to test the account that uncertainty drives decision-making. However, analyzing the data, we found that the uncertainty ratings that people give are confounded with the variability (i.e., riskiness) of the outcomes of the option that the participants chose. Thus, participants tend to give higher uncertainty ratings whenever they choose the risky option, all else being equal (see plots below). Note also that participants differ in this tendency, which makes the issue even more complex. In our opinion, this means that uncertainty ratings are not a good measure of neurocognitive noise, but more likely a mixture of different latent cognitive variables. These issues have recently been formalised in a more general discussion on uncertainty and perceptual decision-making (e.g., Navajas et al., 2017, Nature Human Behavior). Thus, our measure of self-reported uncertainty ratings is difficult to interpret and cannot be linked to cognitive noise alone. Since this debate is not central to our main message, and since our paper already contains a lot of data as well as both cognitive and neurocomputational models, we chose to not describe these uncertainty rating data. However, if the reviewer believes these data will strengthen the paper (rather than distract from the core findings), we could still include them, with the necessary caveats regarding their interpretation.

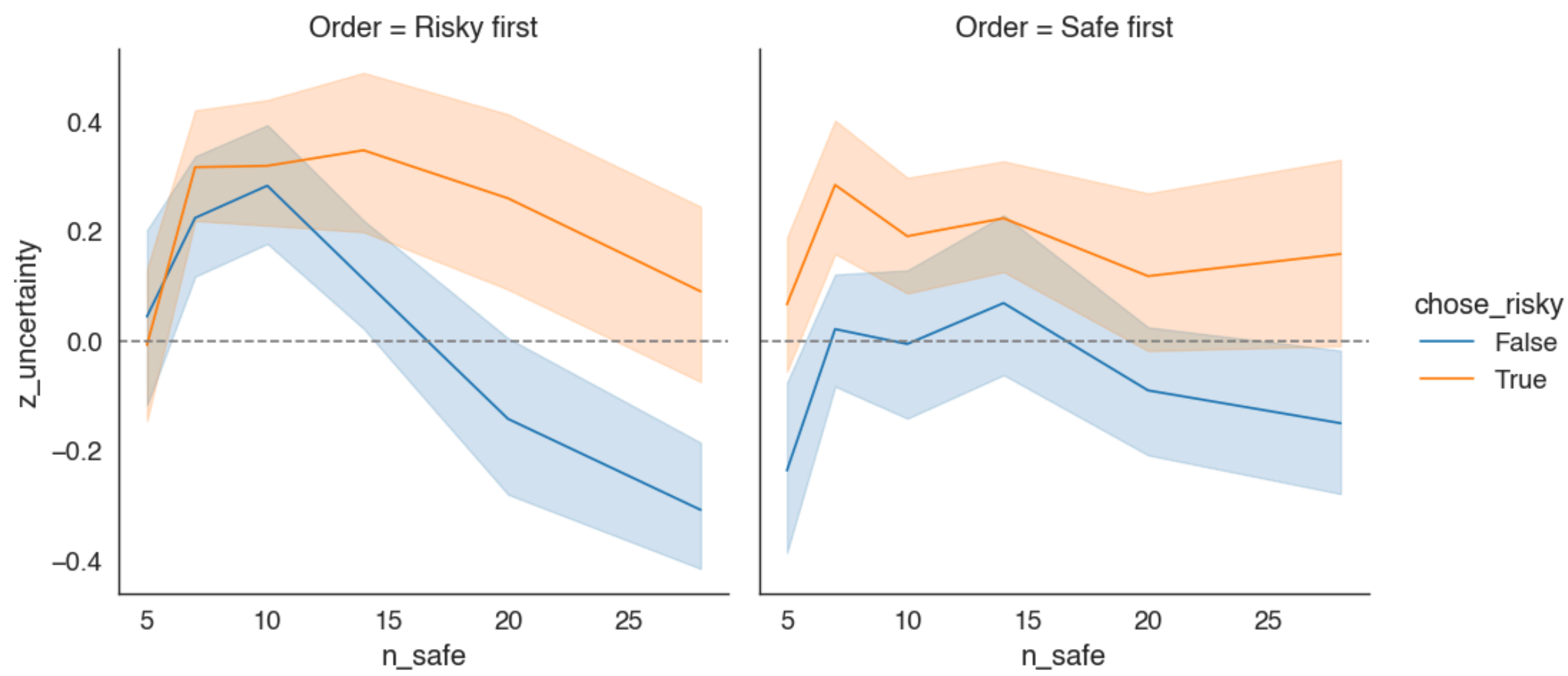

Quick illustration: participants always report higher uncertainty when they chose the riskier option, independent of stake sizes.

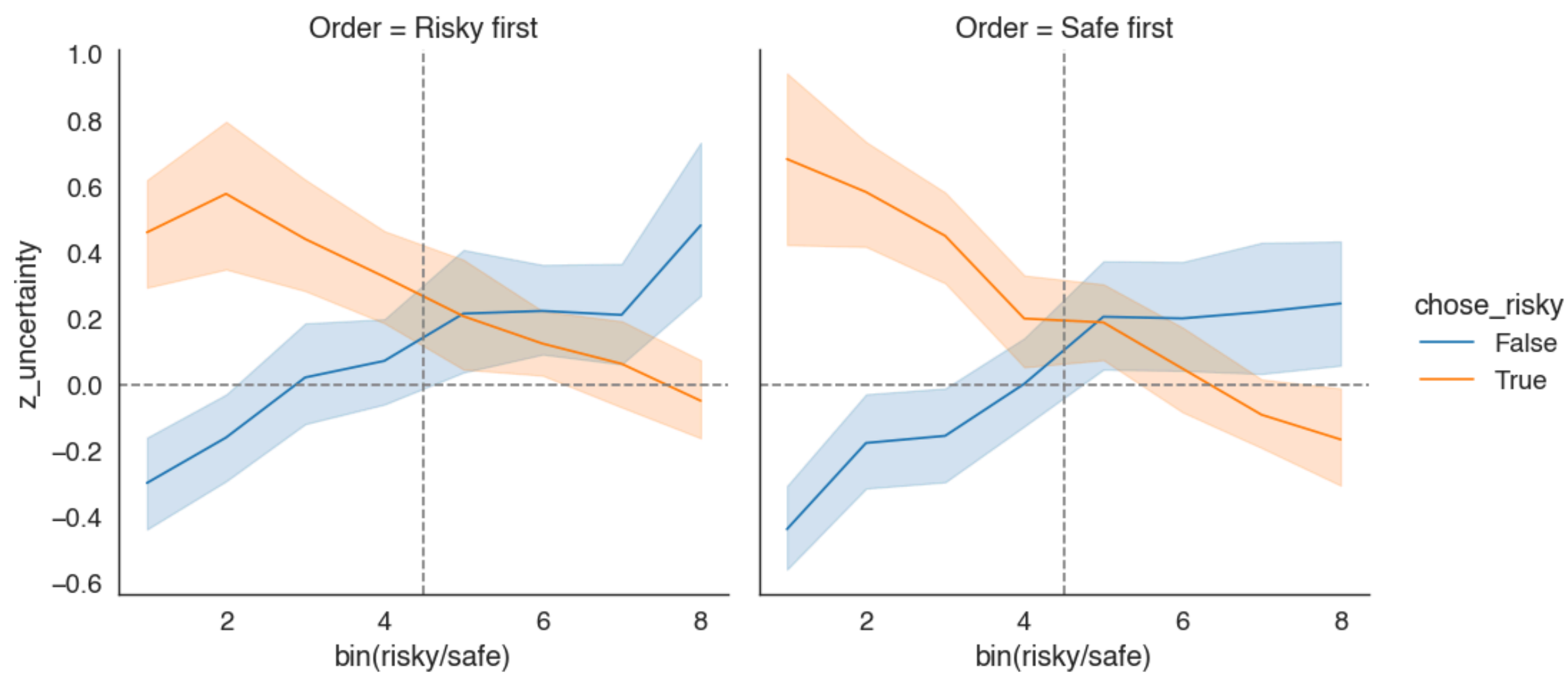

Another quick illustration: Participants generally report higher uncertainty when they chose the riskier option, over and above the effect of the ratio of the payoffs of the risky and safe option (i.e., the orange and blue curve are not symmetric around .5).

In closing, we also thank this reviewer for the many helpful comments and suggestions, which have helped us strengthen our paper.

Point-by-Point Response to Reviewers

"Rapid Changes in Risk Attitudes Originate from Bayesian Inference on Parietal Magnitude Representations"

Nature Communications — Round 2 Revision

Reviewer comments are reproduced verbatim in italics. Our responses follow in plain text.

<u>Reviewer 1</u>

> *The authors have addressed my comments from the last round. The framing of the paper and its contribution is much-improved. I want to emphasize that I think this is an excellent study that will make an exciting contribution to the literature on choice under uncertainty. While the existence of sequential presentation effects on risky choice are known in the literature, this paper provides both a rigorous theoretical mechanism as well as additional process data (numerical perception derived from fMRI of partial cortex) to justify the argument that variation in numerical perception is linked to choice behaviour. The array of methods and data sources are impressive and compelling. As noted in my last review, Figure 4 is a highlight and is an important contribution to our understanding of risky choice behaviour and how to measure risk preferences. While my comments with respect to framing may seem critical, they are intended to improve the paper so that it has maximum impact across a wide range of fields (including economic theory). I hope they are interpreted as an attempt to improve the paper for publication in Nature Communications. I have a few remaining comments, detailed below.*

We thank Reviewer 1 for their generous and thoughtful assessment. We are delighted that they consider this an excellent study and that they find the revisions from the previous round satisfactory. We address their remaining comments below.

Comment 1

> *The addition of the behavioural task using Arabic numerals improves the paper and will help justify its conclusions for risky choice more broadly. I would suggest emphasizing this task, and the implication of its results, earlier in the introduction (lines 145–157). Currently this paragraph defends the use of the numerosity clouds to justify the fMRI experiment. All well and good. But a sentence here noting that the numeral task exists and that the behaviour was analyzed (and similar) across both tasks would help the reader place the following results in context. Currently, it's not until lines 236 that we learn that task even exists, and lines 570–590 that the behaviour is similar!*

We thank the reviewer for this helpful suggestion. We have revised lines 145–157 of the introduction to include an explicit forward reference to the Arabic numeral task and to note upfront that the key behavioural signatures are consistent across both task variants. We agree that this substantially improves the narrative flow and contextualises the subsequent results for a broader readership. We have now revised the manuscript as follows: [L157 - 161]

> *In the following, we will first describe the risky choice paradigm we developed to modulate the acuity with which choice options are represented in the brain, by manipulating the order in which they are presented. This experimental paradigm also allows us to extract an fMRI signal that corresponds specifically to the neural acuity of the perception of the payoff of the first choice option, independent from any choice-related neural activity that comes later in the trial[35]. To be able to do so, we used the numerical magnitudes of dot clouds to present the payoff magnitudes to the participants, as we have in earlier work[35]. Such stimuli are somewhat different from the Arab numerals often employed to present potential payoffs; however, seminal work in numerical cognition has established that the neural processing of the numerosity of such stimuli is similar to that of more general (symbolic) numerical information[37,38], including Arab numerals[35]. Numerosity processing for both types of stimuli engages a more general parietal 'magnitude system'[39], and the acuity of numerosity processing for both types of stimuli is correlated across individuals[35]. However, to establish that our findings are not specific to dot cloud stimuli, we also administered a behavioural version of the task in which payoff magnitudes were presented as Arab numerals. The key behavioural effects were consistent across both task variants, demonstrating that the observed changes in risk attitudes generalise across numerosity display formats.*

Comment 2

> *lines 84-90 In reference to Random Utility and recent advances in economic theory, the authors write: "Although these theories alleviate the problem of choice inconsistency, they do not address the question of where this randomness comes from, or why risk attitudes are so dependent on context (e.g., order of presentation). Both theories frame the noise in observed behaviour as random and therefore external to the decision-making processes dealing with the choice-relevant information (e.g., payoffs). This stands in stark contrast to numerous findings in perceptual neuroscience that the degree of randomness in observed choices is usually tightly linked to the amount of bias in decisions, suggesting a common origin. In contrast to RUT, more recent economic choice models attribute risk attitudes directly to the noisiness of how decision-makers perceive the choice problems they are faced with." This is not quite accurately framed. First, I agree with the author's main point that we need models in which the degree of randomness is linked to the amount of bias, and that the source of this randomness may be perceptual (or at least related to information acquisition). However it is not clear that this lies "In contrast to RUT..." rather these models may lie inside this class.*

There are two theoretical issues here. First, to my knowledge it is not known whether an efficient coding model is, or is not, an RU model. It is important to note that the class of RU models is quite "large" in the sense that many other theories/models fall in this class. This is because the necessary and sufficient conditions for RU are rather weak (in terms of the restrictions they place on choice behaviour). A recent empirical example is given by McCausland et al. (2020). I believe the authors have in mind a specific version of a RU model in which stochastic utilities are assumed to be exogenous, independent of context, and from a narrow class of distributions. This model _does_ place stronger restrictions on behaviour. But that is only a subset of models in the class of RU. One simple example is violations of IIA, some of which RU can account for through the choice of error distribution. I am not aware of any theoretical demonstration that efficient coding models violate regularity (a necessary condition for RU). Rather, a more plausible conjecture is that efficient coding models fall inside the class of RU. If one interprets the encoding function m() as a utility functon(as Netzer et al. do), then efficient coding models relax the requirement that the utility function depends on context. This is most apparent in Payzan-LeNestour & Woodford (Fig 2) where they describe a simple efficient coding model on a single outcome dimension. The probability of an error is given by the difference in the means of the posterior (i.e. the difference in the utility function m()), just as in random utility. Except now the utility function and error distribution are jointly (optimally) chosen via the principles of efficient coding. This is the key contribution of efficient coding because the general class of random utility models do not necessarily impose this requirement (i.e. while not necessary, it is sufficient). Because the model described by LeNestour & Woodford falls under the class of random utility, it would not be accurate to say that efficient coding is not random utility. For the author's statement to be true, efficient coding models would need to violate the Block-Marshak inequalities (necessary and sufficient conditions for RU), and it is not obzvious to me that they do. If the authors can justify this statement, then that is an important contribution in its own right. Note that the above argument provides sufficient motivation to study the author's question because we want to understand the efficient coding function, m(), how it depends on context, and what it implies for measuring risk preferences.

Second, there is another strain of theoretical literature which makes this point about random utility as well. Fosgerau et al. demonstrate an equivalence between Random Utility and Rational Inattention, such that any RU model has a RI interpretation. In a RI model, the noise in decisions arises from the agent optically selecting (or ignoring) information because of constraints/costs on information processing (Mackowiak et al.). See also Hebert and Woodford on dynamic information acquisition. Therefore the stochasticity in RU can be interpreted as arising from from optimal information acquisition and can depend on the context of the problem (i.e. which information is costly, and which payoffs

are worth collecting information about). This falsifies the final sentence in the author's paragraph. Moreover, some of these findings are only a few years old, so would also qualify as "recent". To my knowledge, it is not known how RI models map on to efficient coding models (though the discussion above would suggest there is some overlap). Again, this is sufficient motivation to examine efficient coding models in the context of the author's study.

We thank the reviewer for this careful and important correction. We agree that our original framing could have been understood as too strong a statement. We have revised the relevant passage to remove the claim that efficient coding models stand "in contrast to" Random Utility Theory altogether. Instead, we now present these models simply as being inspired by the perceptual neuroscience literature, making no claim about their relationship to the broader class of RU models. Specifically, we revised the manuscript as follows [L81 - 92]:

Adaptations of the preferential framework have tried to account for the stochasticity of risky choice by adding a random noise term to the utility function (Random Utility Theory, RUT)[18] or by assuming that decision-makers may have a preference for responding randomly[19]. Although these theories alleviate the problem of choice inconsistency, they do not specifically address the question of where this randomness comes from, and generally do not explain why risk attitudes are so dependent on context (e.g, order of presentation). By contrast, numerous findings in perceptual neuroscience suggest that the degree of randomness in perception is tightly linked to the amount of bias in decisions, suggesting a common origin in perceptual noise[20–26]. This raises the possibility that the noisiness of perception might directly underlie also some aspects of economic attitudes. In line with this possibility, some models of economic choice, inspired by the perceptual neuroscience literature[16], attribute risk attitudes directly to the noisiness of how decision-makers perceive the choice problems they are faced with[27–31].

Minor Issues

What is "model stacking" — it is mentioned a few times throughout the paper but not described.

We have added a brief definition the first time "model stacking" appears in the manuscript ([L268 - 271]). Specifically, we revised the sentence to read: "formal model comparisons revealed that the full interaction model received a model weight of 95% using model stacking (a method that finds the optimal linear combination of competing models based on their leave-one-out predictive accuracy[48,49])," where references 48 and 49 refer to Yao et al. (2018) and Kumar et al. (2019) respectively.

Line 666 — "…evidently…" — do the authors mean "obviously" here instead?

We have replaced "evidently" with "obviously" at line 666, as suggested.

Reviewer 2

I appreciate the authors' efforts to address my concerns and questions.

We thank Reviewer 2 for their continued engagement. We note that Reviewer 2 and the authors hold a persistent disagreement about the conceptual framing of the task. We have done our best to address this clearly and precisely below.

Comment 1

Regarding my main concern---namely, what the task actually measures---I disagree with the authors' claim that the task measures risk preference simply because it includes a risky option on each trial. Because of how reward magnitudes are presented and because reward probability of the risky option, which is supposed to introduce risk, is fixed across trials, the task does not meaningfully manipulate risk. Because probabilities are fixed, they can be absorbed into outcome evaluation, as suggested by Prospect Theory and other heuristic models of risky choice, reducing the task to a comparison of reward magnitudes. The issue is therefore not the gap between theoretical and observable manifestations of risk attitude, but rather the absence of any manipulation of risk (i.e., probabilities) in the first place. As implemented, the task is too simplistic to reveal anything about risk preference, which is why the findings and framing focus entirely on the representation of reward magnitude, with no real link to risk preference. Changing reward probabilities is not strictly required to measure risk preference, but in the current experiment, with the probability of the risky option held constant, the task effectively reduces to comparing reward magnitudes in terms of expected outcomes. Moreover, as many have noted, risk aversion in Expected Utility Theory is not truly about risk in the sense of probability processing, but rather about how reward magnitude is processed. Prospect Theory is the framework that meaningfully addresses risk preference by incorporating probability distortion, which is entirely ignored here. In this context, it is unclear how one can propose a model of risk preference reversal when probability does not enter the model at all and everything depends on the representation of payoffs. In another word, probability does not play a role in the observed behavior; the task involves comparisons of magnitudes. As such, the study is fundamentally about magnitude representation in a choice context involving a sure and a risky option, rather than about risk preference itself. One could replace the

probability dimension with another dimension, such as effort (for example, choosing between two options that offer different reward magnitudes and differ by a fixed amount of effort), without changing the core results.

We appreciate the reviewer's continued engagement on this point. However, we respectfully maintain our original position for the following reasons.

First, we note that the terminology in our manuscript was substantially revised following Round 1 peer review, precisely to address concerns of this kind. We now consistently distinguish between risk preferences (latent, defined by utility function curvature, and not directly observable) and risk attitudes (overt choice behaviour, which serves as the standard empirical measure via which preferences are inferred). Our task measures the latter: we observe how participants choose between safe and risky options, and we characterise the resulting behavioural patterns — that is exactly what "risk attitudes" means in our usage, and exactly what the task is designed to measure. We do not claim that our task directly measures the underlying latent preferences, and the manuscript is explicit on this point.

Second, within this framework, the relevant question is not whether our task varies probability across trials, but whether it elicits the choice behaviour through which risk attitudes are expressed. In Expected Utility Theory, a risk-averse attitude is defined by concavity of the utility function over outcomes — the definition formalised by Pratt (1964). Rothschild & Stiglitz (1970) then proved this to be equivalent to preferring less variable options: formally, EU(X) > EU(Y) whenever X is less variable than Y in the sense of a mean-preserving spread (their Theorem 2). This definition does not require probability to vary across trials to be expressed or measured. A participant who systematically reveals a behavioural preference for the safe option over a higher-EV risky option is exhibiting risk aversion in exactly this sense: the safe option is less variable, and the systematic revealed choice of it over the riskier alternative is, by the Pratt–Rothschild–Stiglitz definition, the behavioural signature of a risk-averse attitude. Fixed-probability paradigms elicit precisely this behavioural signature and are widely used in influential studies of risk attitudes for this reason, including Tom et al. (2007), Khaw, Li & Woodford (2020), and Schneider et al. (2026). We therefore respectfully maintain that our task is fully in line with measures of risk attitudes, both in the classic and contemporary literature.

Third, we would note that the reviewer's two additional arguments in fact support rather than undermine our conclusions. The claim that fixed probabilities reduce the task to "a comparison of reward magnitudes" is precisely what we demonstrate as a central finding of our manuscript: observed risk attitudes in laboratory tasks are substantially influenced by properties of neuro-cognitive magnitude representations. Similarly, the suggestion that the probability dimension could be replaced by another dimension such as effort without changing the core results would, if correct, only strengthen this point — it would demonstrate that what is commonly labelled "risk

preference" in such tasks is not specific to risk, but reflects properties of perceptual representation of choice-relevant magnitudes. We consider this a core conclusion of our study, not a limitation of our paradigm.

Fourth, the reviewer argues that Prospect Theory — with its emphasis on probability distortion — is the correct framework for measuring risk preference. We respectfully disagree that probability distortion is necessary for a task to elicit risk attitudes. Our study is explicitly situated within the EUT tradition, in which risk attitude arises from the shape of the utility function over magnitudes. The absence of probability variation is a deliberate design feature, not a limitation: it allows us to isolate the contribution of magnitude representation to choice behaviour, which is precisely our research question.

Finally, we note that Reviewer 2 themselves writes: "I agree with the authors that their proposed Bayesian model provides plausible mechanisms for the observed behavior, namely asymmetric processing of reward magnitude depending on order." We would point out that observed behavior in risky choice tasks is, by definition, what the field calls risk attitudes — the overt behavioural expression of underlying latent preferences, which cannot themselves be measured directly. The reviewer thus appears to agree with the core empirical and modelling contribution, while disputing only how that observed behaviour should be labelled. This is precisely the terminological distinction — between latent risk preferences and overt risk attitudes — that was already introduced in the Round 1 revision and that the manuscript now consistently employs. We therefore believe the manuscript as currently written directly addresses this concern, and we hope the above (and the changes to the manuscript) further clarify our position.

Comment 2

> *Regarding Figure 1C, my point was that within a given level of stakes, the probability of choosing the risky option is relatively similar across different payoff ratios. There is a small interaction effect for the "small stakes" condition, most likely because participants can count the dots, which largely eliminates the order effect. I am glad the authors agree that what they observe is a perceptual bias, but as I argue above, this does not link to risk preference. If reward magnitude is the only variable subjects process in this task, then any change in preference reflects changes in the representation of reward magnitude, not changes in risk preference. Naturally, a noisier representation of reward magnitude will directly translate into noisier behavior when there are no other factors that change across trials.*

We thank the reviewer for this comment. We agree that the order effect reflects a perceptual bias in the representation of reward magnitude — indeed, this is the central

claim of our paper. However, we respectfully note that this is precisely the point: when observed risk attitudes (choices between safe and risky options) can be driven by changes in magnitude representation, this demonstrates that what is commonly measured as a "change in risk preference" in laboratory experiments can in fact reflect a perceptual phenomenon. The reviewer's observation therefore does not undermine our conclusions at all, but confirms what we set out to show with our study. Thus, the reviewer's restatement of our central finding — that systematic changes in choice behaviour can reflect changes in neurocognitive magnitude representation rather than changes in latent risk preferences — is, in our view, an accurate and concise summary of our paper's contribution.

Comment 3

> *A more appropriate reference for different types of models is Dayan and Abbott (2001), while Abbott (2001) also addresses relevant points. That said, I agree with the authors that their proposed Bayesian model provides plausible mechanisms for the observed behavior, namely asymmetric processing of reward magnitude depending on order.*

We thank the reviewer for this suggestion. However, Dayan & Abbott (2001) is a textbook covering a broad range of topics in theoretical neuroscience, and the reviewer has not specified which chapter or section they consider relevant to our work. We welcome clarification on this point and would be happy to add more specific pointers, but in the absence of such pointers, we have not added a reference to the whole book.

Comment 4

> *With respect to the new uncertainty-related analysis, I first thank the authors for including an analysis of the uncertainty reports. However, I am disappointed that the uncertainty reports do not reflect the order effect, which the authors attribute to noise in encoding that supposedly underlies risk preference. This result ultimately undermines their conclusions.*

We respectfully disagree with this conclusion. It is well established in the cognitive science literature that subjective confidence reports are not equivalent to, and do not directly index, internal epistemic uncertainty (Fleming & Dolan 2012; Pouget et al. 2016). Confidence judgments are influenced by many factors extraneous to the primary decision, including the riskiness of the chosen option, post-decisional processes, and idiosyncratic response biases. In our paradigm, there is strong evidence that participants interpret the confidence question partly in terms of whether they chose the

risky option, reporting lower confidence simply because they took a gamble with an uncertain outcome. This would obviously mask any order-related uncertainty signal on the confidence scale. Thus, the absence of order effects in the confidence judgements reflects a well-documented limitation of self-report confidence as a proxy for internal noise, not a failure of our model or conclusions about the origins of risk attitudes.

In closing, we thank the reviewers for evaluating our manuscript and providing comments that have helped us strengthen the paper. We hope the revised version now addresses all outstanding concerns and is now ready for publication.

Dear Editor,

Thank you for the decision to accept our manuscript in principle for publication in Nature Communications. We are grateful to you and to both reviewers for the constructive engagement throughout the review process.

We have revised the manuscript to address the editorial requests in the Author Checklist, together with the remaining comments from the reviewers. Reviewer 1 recommends acceptance and suggested a single minor wording change, which we have implemented. Reviewer 2 did not request any new changes but restated their position; we respond to each point below.

In what follows, the reviewers' comments are reproduced in full and in italics, with our responses in plain text.

Reviewer #1 (Remarks to the Author)

Thank you to the authors for the latest revisions. I think this is an excellent paper with an impactful contribution to the literature on choice under uncertainty. My recommendation is to accept the manuscript.

With that said, I do I think it may be useful to clarify some confusion in the review process. I am not asking for any changes to be made to the manuscript unless the authors wish to include them, rather this point is simply made with the hope that it resolves further debate.

1) The textbook definition of risk aversion requires only the observation of a single choice of a single lottery. Below is Definition 6.C.1 in Mas-Collel, Whinston, and Green.

"A preference relation over lotteries is risk averse if, for any lottery L, the degenerate lottery that yields the expected value of L with probability one is weakly preferred to L itself."

Note the "for any lottery L" condition, it is not "for every" or for "multiple", only one lottery is needed. Therefore in the author's experiment, the behavioural observation of "risk aversion," as typically defined in economics, can be inferred from a choice between a single 50/50 lottery and a sure thing. Therefore the author's experiment is valid for measuring risk preferences as defined in economic theory.

The authors do not attempt to manipulate "risk" in their experiment. Rather, they demonstrate that the sequencing of lottery information can alter the lottery choice behaviour of subjects. This is the important insight of the paper!! By the definition of risk aversion noted above, this means that the standard behavioural observation used to identify "risk preference" is fraught, and that choice is therefore not sufficient to identify preferences. A similar theoretical point is made by Netzer et al., (2025) in which an agent with risk neutral preferences will behave in a manner that "appears risk averse". While previous studies have demonstrated manipulations which alter lottery choice behaviour, this study links the manipulation directly to numerical processing in the brain with a clear neuro-psychological mechanism. Therefore, as demonstrated by the authors, we should not infer risk preferences from a single 50/50 lottery due to noisy magnitude representation. This means that the field of economics needs to reconsider one of its key tenets: the link between behaviour and preferences. Assessing the predictions of Prospect Theory, and the subsequent "fourfold

pattern," requires an experiment that manipulates probabilities. However the authors are not making claims about the broader collection of empirical results that are covered by Prospect Theory.

References

Mas-Collel, A., Whinston, M. D. & Green, J. Microeconomic Theory. (Oxford University Press, 1995).

Response. We thank the reviewer for recommending acceptance and for this clarification, which we agree resolves the central point of debate. The textbook definition of risk aversion (Mas-Colell, Whinston & Green, Def. 6.C.1) requires only a single choice between a lottery and a sure thing—precisely the choice our participants make—and does not require manipulating probabilities. We share the reviewer's reading that the contribution of our study is exactly to show that this canonical behavioural observation is not sufficient to identify preferences, because the same choice can be shifted by how the payoff magnitudes are perceived and represented. As the reviewer notes that no changes are required, we have left this framing unchanged and have implemented only some small clarifications in the text, plus their minor suggestion below.

> *While this evidently does not prove that subjective valuation is not involved in risky choices, it still suggests that perception may play an important role and needs to be accounted for explicitly when researchers attempt to measure risk preferences, at least in the small-stake laboratory experiments widely used in the field.*
>
> *Future work in this direction may, for example, link economic preferences as inferred from choice behavior to the noisiness of neural representations of other relevant economic choice variables, like probability[72,96,101], time[105,106], and social constellations[107,108]. This may further our mechanistic understanding of how these variables are systematically incorporated into choice and should thus inform further development of the corresponding economic models[4,42,107,109–111].*

Minor

Change "adding a random noise term to the utility function" to "assume that preferences are stochastic". The first phrase describes only a subset of RU models which assume an additive random component, this is not the full set of RU models as outlined in the last revision note.

Response. We have made this change. The sentence in the Introduction now reads:

> *"Adaptations of the preferential framework have tried to account for the stochasticity of risky choice by assuming that preferences are stochastic (Random Utility Theory, RUT) or by assuming that decision-makers may have a preference for responding randomly."*

Reviewer #2 (Remarks to the Author)

Comment 1:

I am not persuaded by the authors' claim that their task measures risk attitude simply because one of the options is probabilistic, for the reasons I outlined above. In addition, the distinction they draw between "risk preference" and "risk attitude" is completely arbitrary. These terms are often used interchangeably, and when a difference is made, it is typically that "risk attitude" refers to more innate or stable tendencies, whereas "risk preference" emphasizes the more flexible, context-dependent aspects of risky decision-making (see, for example, Chapter 9 of the Neuroeconomics volume edited by Glimcher and Fehr).

In addition, although expected utility theory (EUT) "captures" risk aversion through a concave utility function, it does not truly explain risk preference, because it is not explicitly concerned with probability processing. The fact that some studies still rely on an outdated approach in which reward probability is not manipulated to study risk preference does not justify this practice. One can certainly observe the effects of risk preference using an option with probabilisitic outcome, but without systematically varying the key component of risk—reward probability—it is impossible to measure how probability processing contributes to behavior. This is why, in my view, the current study is effectively reduced to evaluating reward magnitude, with little or no connection to genuine risk preference.

Comment 2:

I completely disagree with the authors' interpretation and feel they are largely playing with terminology to support their claims. If the task manipulates only reward magnitude and keeps reward probability fixed, then it is not measuring risk preference in any meaningful sense, since risk preference—by standard definitions—concerns choices over uncertain outcomes that explicitly involve probability weighting and probability-sensitive tradeoffs.

Response (Comments 1 and 2). We have addressed these points in detail across the previous rounds and in the corresponding revisions, and the reviewer requests no new changes here, so we briefly reaffirm our position. A choice between a sure amount and a lottery of equal or comparable expected value is the canonical operationalization of a risky choice in economic theory, and it does not require varying probabilities across trials. This follows directly from the textbook definition of risk aversion reiterated by Reviewer 1 (Mas-Colell et al., Def. 6.C.1), as well as from the foundational treatments of risk aversion in Pratt (1964) and of "increasing risk" as a mean-preserving spread in Rothschild & Stiglitz (1970)—none of which invoke manipulation of probabilities. Expected utility theory, the standard first approximation to risky choice, contains no probability distortion and is fully interpretable with fixed probabilities. We are explicit that we do not study probability weighting and make no claims about the fourfold pattern of prospect theory; probabilities were held fixed deliberately to keep the neurocomputational modeling tractable and to maximize statistical power for the effects of interest, as now stated in the manuscript.

Regarding terminology: conventions for "risk preference" versus "risk attitude" do indeed differ across literatures, which is exactly why we define both terms explicitly in the Introduction—risk preference as a latent theoretical construct, and risk attitude as its observable manifestation in choice. Crucially, our empirical result does not depend on this

labelling: the directly measured tendency to choose the sure option shifts systematically with presentation order and stake, and this shift tracks trial-to-trial neural representational noise. That finding stands regardless of which term one assigns to the latent versus the observed quantity.

Comment 3:

As I mentioned earlier, there is no disagreement between us regarding what the Bayesian model contributes.

Response. We thank the reviewer; we are glad there is agreement on the contribution of the Bayesian model.

Comment 4:

I maintain my position that the confidence results undermine the central claim of this study.

Response. As discussed previously, the self-reported uncertainty ratings are confounded with the riskiness of the chosen option—participants report higher uncertainty whenever they choose the risky option, over and above the payoff ratio—and this tendency itself varies across participants. The ratings therefore reflect a mixture of latent variables rather than a clean readout of neurocognitive noise, and they do not bear on the central claim, which rests on the behavioral and neural modeling. We accordingly maintain our interpretation.

We thank the editor and both reviewers once more for their time and for helping us improve the manuscript, and we look forward to seeing it published.

Sincerely,

Gilles de Hollander
on behalf of all authors (Gilles de Hollander, Marcus Grueschow, Franciszek Hennel, Christian C. Ruff)
